# Microbiome assembly predictably shapes diversity across a range of disturbance frequencies in experimental microcosms

**DOI:** 10.1038/s41522-022-00301-3

**Published:** 2022-05-13

**Authors:** Ezequiel Santillan, Stefan Wuertz

**Affiliations:** 1 Singapore Centre for Environmental Life Sciences Engineering, Nanyang Technological University Singapore, Singapore, 637551 Singapore; 2 School of Civil and Environmental Engineering, Nanyang Technological University Singapore, Singapore, 639798 Singapore

**Keywords:** Next-generation sequencing, Bacteria, Microbial ecology, Water microbiology

## Abstract

Diversity is often implied to have a positive effect on the functional stability of ecological communities. However, its relationship with stochastic and deterministic assembly mechanisms remains largely unknown, particularly under fluctuating disturbances. Here, we subjected complex bacterial communities in microcosms to different frequencies of alteration in substrate feeding scheme, tracking temporal dynamics in their assembly, structure and function. Activated sludge bioreactors were subjected to six different frequencies of double organic loading, either never (undisturbed), every 8, 6, 4, or 2 days (intermediately disturbed), or every day (press disturbed), and operated in daily cycles for 42 days. Null modeling revealed a stronger role of stochastic assembly at intermediate disturbance frequencies, with a peak in stochasticity that preceded the occurrence of a peak in α-diversity. Communities at extreme ends of the disturbance range had the lowest α-diversity and highest within-treatment similarity in terms of β-diversity, with stronger deterministic assembly. Increased carbon removal and microbial aggregate settleability (general functions) correlated with stronger deterministic processes. In contrast, higher stochasticity correlated with higher nitrogen removal (a specialized function) only during initial successional stages at intermediate disturbance frequencies. We show that changes in assembly processes predictably precede changes in diversity under a gradient of disturbance frequencies, advancing our understanding of the mechanisms behind disturbance–diversity–function relationships.

## Introduction

Microbes typically exist as diverse, complex and dynamic communities^1^ and are involved in all biogeochemical cycles^2^. These microbial communities or microbiomes provide crucial functions for global climate regulation, human health, biotechnology and bioremediation^3^. Microbial diversity is often related to community function^4^ and the ability to withstand environmental fluctuations that typically occur as disturbances^5^. Disturbance can be defined as an event in time that disrupts the structure of a community by changing resources, substrate availability, or the physical environment^6^. When disturbance is continuous, it is categorized as press disturbance^5^. While a disturbance may result in inhibition, injury, or death for some individuals in a community, it also creates opportunities for other individuals to grow or reproduce^7^. Indeed, disturbance is considered a major factor influencing species diversity^6^, but a clear understanding of the underlying mechanisms is lacking^8,9^.

Given the growing human population and its impact on natural and engineered ecosystems^10^, management and conservation practices are faced with increasing frequencies and magnitudes of various disturbances that occur on different scales. A concept of ecology that can be used to explore possible outcomes is the intermediate disturbance hypothesis (IDH), which predicts a diversity peak at intermediate levels of disturbance due to competition-colonization trade-offs faced by organisms^11^. The IDH has been influential in ecology^12^ and ecosystem conservation^13,14^. However, it is not a coexistence mechanism as initially thought, but rather a family of spatial and/or temporal processes resulting in higher diversity under intermediate disturbances^15^. Further, many studies have not found the diversity pattern predicted by the IDH^16,17^ and its relevance as a prediction tool is up for debate^18,19^. Therefore, studies are needed to address the mechanisms behind the observed disturbance–diversity relationships^20^.

Community assembly processes are believed to shape community structure^21^, which also links them to ecosystem function. These processes can be either deterministic, when communities form due to selection imposed by abiotic or biotic factors^22^, or stochastic, assuming that all taxa have a similar fitness and the structure of the community is shaped by random events of ecological drift (i.e., births and deaths)^23^. Both deterministic and stochastic processes are known to simultaneously influence the assembly of communities^24–27^. Although disturbance is believed to be an important driver of community assembly processes^28^, its effects on their relative importance are not well understood^29^. Disturbance can elicit stochastic assembly mechanisms that lead communities to different states of structure and function^9,30^, despite using replicated experimental settings^31^. Further, while recent studies have reported positive correlations of strength of stochasticity with α-diversity in bacterial^32^ and fungal^33^ communities, the role of assembly processes behind diversity patterns under fluctuating disturbances is still unclear.

Intermediate frequencies of exposure to a xenobiotic pollutant (3-chloroaniline) in our recent replicated sludge bioreactor study demonstrated higher α-diversity and relative influence of stochastic assembly compared to other exposure levels, after a succession period of 35 days^9^. We hypothesized that when intermediate disturbance frequencies gave rise to unpredictable environments for organisms rendering their specialized traits less advantageous, stochastic equalization of competitive advantages across the overall pool of organisms would lead to a higher α-diversity. In contrast, either no disturbance or press disturbance conditions at the extreme ends of a disturbance range would allow fewer adapted organisms to dominate, thus lowering the α-diversity. We named this causal relationship the intermediate stochasticity hypothesis (ISH)^9^, which could also be framed as an intermediate disturbance-maximum stochasticity-and-diversity hypothesis. Unlike the IDH, the ISH incorporates assembly mechanisms that shape community structure (α- and β-diversity) across a disturbance gradient. Further, it predicts not only a pattern in species richness, as originally conceived in the IDH, but also in higher-order α-diversity indices since variations in the underlying assembly mechanisms would also affect the abundance distributions of taxa. The ISH further considers that the output of a stochastic process is affected by some uncertainty, which in this case means there are several possible paths for the evolution of the structure and function of a community. In this regard, stochasticity operating at intermediate levels of disturbance in replicated systems could lead to similar high α-diversity (local, e.g., within a reactor), but not necessarily to similar β-diversity (compositional variation across sites, *e.g*., between reactors) and community function^9^. However, more research is needed to test the broad validity of the ISH since disturbance is a multidimensional phenomenon^8^, as it can be of different types and have different frequencies, intensities, and extents^34^.

The objective of this work was to test the central tenet of the ISH that intermediate disturbance frequencies promote stochastic assembly processes, resulting in increased α-diversity and variable β-diversity^9^. We used an experimental system comprised of activated sludge sequencing batch reactors harboring complex microbial communities collected from a full-scale wastewater treatment plant. These were subjected to different frequencies of alteration in the feeding scheme of the substrate by doubling the organic carbon content in the feed and keeping the nitrogen content constant. Such alteration represents a disturbance for activated sludge systems due to changes in competition for oxygen, substrate, and biofilm space. Indeed, organic loading shocks were shown to affect relevant functions in activated sludge systems, like carbon removal^35^, sludge settleability^36^, and nitrification^37^, as well as the overall structure and assembly of the microbial community^27^. In this study, the reactors had a working volume of 25 mL, representing a microcosm scale^38^. Replicates (*n* = 5) received double organic loading either never (L0, undisturbed), every 8, 6, 4, or 2 days (L1-4, intermediately disturbed), or every day (L5, press disturbed), for 42 days. We tracked temporal dynamics of community assembly, structure and function, without focusing on any particular taxa. Samples were analyzed using 16S rRNA gene metabarcoding and effluent chemical characterization. Patterns of α- and β-diversity were employed to assess temporal dynamics of bacterial community structure. Assembly mechanisms were quantified via null model analysis of phylogenetic turnover for each bioreactor.

## Results

### Intermediate disturbance frequencies exhibit higher taxonomic and phylogenetic α-diversity

**Fig. 1:**
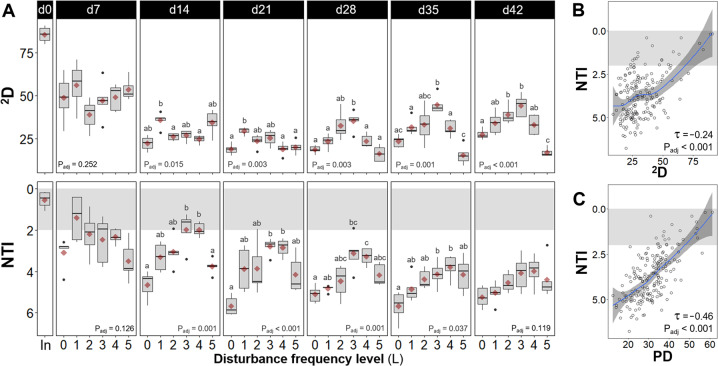
Community dynamics in α-diversity. **A** Community structure assessed via 2nd order Hill α-diversity (^2^D, upper panels) and community assembly evaluated via the nearest taxon index (NTI, lower panels), from bacterial ASV data for different frequencies of organic loading disturbance (*n* = 5). Disturbance frequency levels (L): 0 (undisturbed), 1–4 (intermediately disturbed), 5 (press disturbed). In: sludge inoculum (day 0, *n* = 4). Each panel represents a sampling day, red diamonds display mean values. The box bounds the interquartile range (IQR) divided by the median, and Tukey-style whiskers extend to a maximum of 1.5 times the IQR beyond the box. Characters above boxes display Games-Howell post hoc grouping (*P*_adj_ < 0.05). Welch’s ANOVA *P*-values adjusted at 5% FDR shown within panels. Correlations of **B** ^2^D and **C** phylogenetic diversity (PD) vs. NTI from bacterial ASV data across all frequency levels and time points evaluated in this study (*m* = 184). Kendall correlation *τ*- and adjusted *P*-values are indicated within the panel. Blue line represents locally estimated scatterplot smoothing regression (loess) with confidence interval in dark-gray shading. Note the inverted *y*-axis for NTI, as values closer to zero indicate a higher relative contribution of stochastic assembly. Shaded in gray is the zone of significant stochastic phylogenetic dispersion, |NTI | < 2.

Taxonomic α-diversity was evaluated using Hill diversity indices^39^ of orders zero (^0^D, taxa richness), one (^1^D) and two (^2^D), the latter being a robust estimate of microbial diversity^9^. Phylogenetic α-diversity was also considered through Faith’s phylogenetic distance^40^, both unweighted (PD) and abundance-weighted (PD_W_). There was a temporal decrease in α-diversity for all disturbance frequency levels compared to the sludge inoculum for both taxonomic and phylogenetic α-diversity indices (Fig. 1A and Supplementary Fig. 1). This drop was more pronounced within the first 14 days, when variability across same-level replicates was also highest. A peak in α-diversity at intermediate frequencies of disturbance was observed for all unweighted (^0^D, PD) and abundance-weighted (^1^D, ^2^D, PD_W_) indices evaluated in this study (Fig. 1A and Supplementary Figs. 2 and 3). Such a parabolic pattern was significant from d21 onwards for ^2^D (Welch’s ANOVA *P*_adj_ ≤ 0.003); from d28 onwards for ^1^D (Welch’s ANOVA *P*_adj_ = 0.002–0.01), PD (Welch’s ANOVA *P*_adj_ = 0.003–0.037) and PD_W_ (Welch’s ANOVA *P*_adj_ = 0.005–0.013); and from d35 onwards for ^0^D (Welch’s ANOVA *P*_adj_ = 0.03–0.035).

### Community assembly temporal dynamics precede α-diversity patterns across disturbance frequencies

Assembly processes were first evaluated by modeling the phylogenetic dispersion of a given community against the null expectation, through the nearest taxon index (NTI)^41^. We observed higher stochasticity at the initial stages of the experiment (d0-14), which decreased in relative intensity over time across disturbance levels for both unweighted (ΝΤΙ) and abundance-weighted (ΝΤΙ_W_) indices (Fig. 1A and Supplementary Fig. 2). There was a stronger role of stochastic assembly processes at intermediate disturbance frequencies as shown by ΝΤΙ values closer to zero (i.e., lower | ΝΤΙ| values); this was significant from d14 onward (ΝΤΙ Welch’s ANOVA *P*_adj_ = <0.001–0.037) but was reduced towards the end of the study becoming non-significant on d42. Games-Howell post hoc grouping indicated that the parabolic pattern of ΝΤΙ across disturbance frequency levels preceded (d14-35) the formation of a peak in α-diversity (d21–42) at intermediate levels of disturbance, with two to three groups significantly differentiated (Fig. 1A). Stochastic assembly processes were less prevalent when abundance weighing was included in the calculation of the ΝΤΙ index (ΝΤΙ_W_). This meant that the phylogenetic dispersion of the community, compared to that of the null expectation, was greater when considering the abundance of taxa (i.e., individual organisms) than when only the presence or absence of taxa was considered. Nonetheless, there was a significant peak in ΝΤΙ_W_ values at intermediate frequencies of disturbance on d7 and d14 (ΝΤΙ_W_ Welch’s ANOVA *P*_adj_ = 0.001). This parabolic pattern of ΝΤΙ_W_ was evident on d7, preceding that of ΝΤΙ, but disappeared on d21 and inverted from d28 onwards. Also, significant phylogenetic signals were observed via mantel correlogram analysis (Supplementary Fig. 5) mostly across relatively short phylogenetic distances, justifying the use of phylogenetic null modeling to evaluate community assembly processes in this study.

Stochastic assembly was higher when α-diversity was higher, particularly for phylogenetic diversity. This was shown by significant Kendall correlation *τ* values (−0.24 to −0.46, *P*_adj_ < 0.001) between ΝΤΙ and α-diversity indices (Fig. 1B, C and Supplementary Fig. 4). Kendall correlation *τ* values were also negative (−0.20 to −0.26) and significant (*P*_adj_ < 0.001) between ΝΤΙ_W_ and phylogenetic α-diversity indices (PD, PD_W_) and unweighted taxonomic α-diversity (^0^D), but not between ΝΤΙ_W_ and abundance-weighted taxonomic α-diversity (^1^D, ^2^D) (Supplementary Fig. 4). The estimation of all the aforementioned indices over time using rarefied ASV sequencing data yielded the same significant patterns via Welch’s ANOVA, with the exception of ΝΤΙ_W_ on d21 and d42 (see Supplementary File).

### β-diversity patterns display similarity at low and high disturbance frequencies and higher variability at intermediate ones

**Fig. 2:**
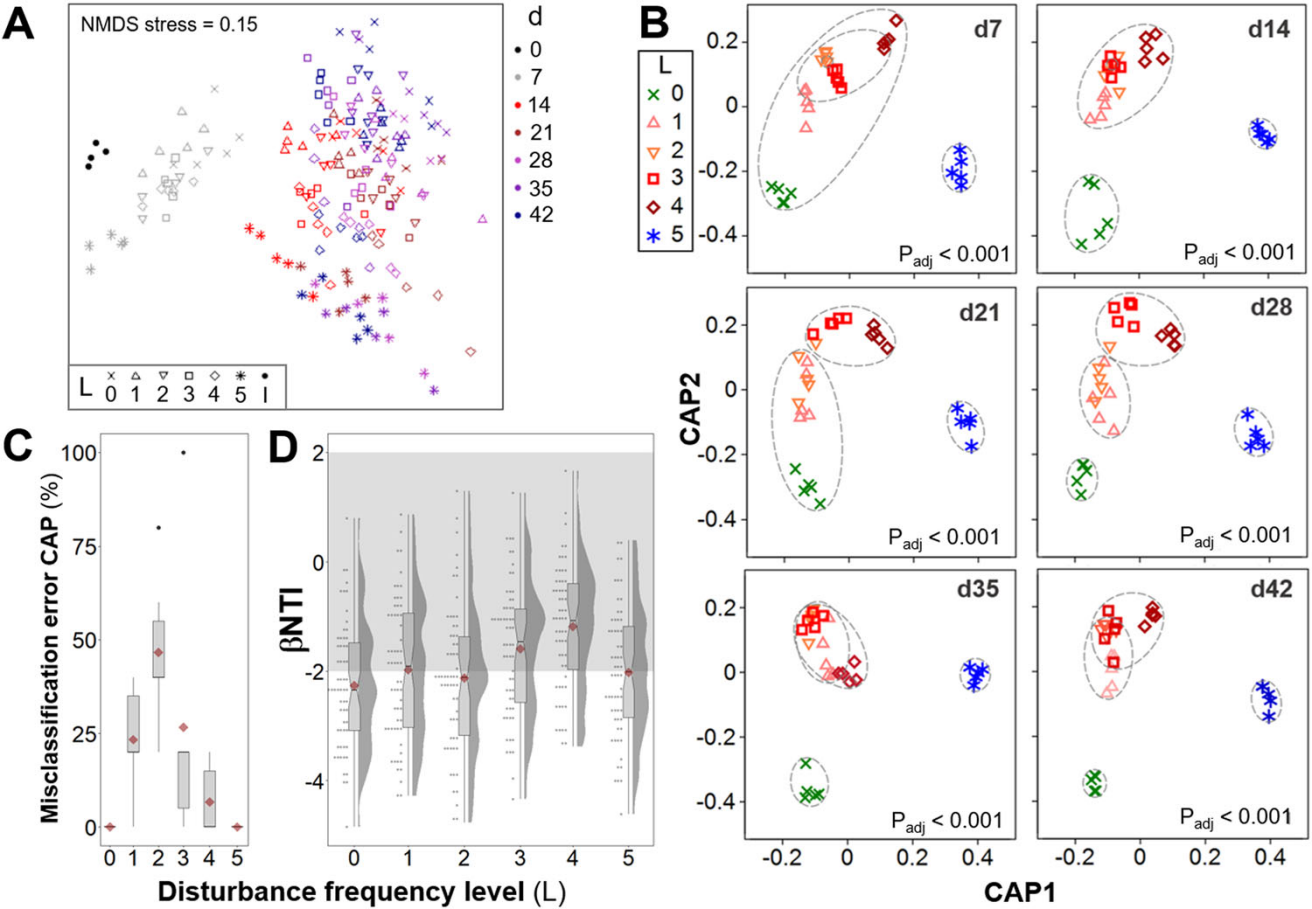
Temporal dynamics of β-diversity community structure and assembly for bacterial ASV data across different frequencies of organic loading disturbance (*n* = 5 bioreactors). **A** Unconstrained NMDS ordination (weighed Unifrac β-diversity, Hellinger transformed data) for all 184 samples collected. Disturbance frequency levels (L): 0 (undisturbed), 1–4 (intermediately disturbed), 5 (press disturbed). I: Sludge inoculum (day 0, *n* = 4). **B** Constrained canonical analysis of principal coordinates (CAP) ordinations (Bray–Curtis β-diversity, squared root transformed data) on different sampling days, including ellipses of 60% group-average cluster similarity and PERMANOVA adjusted *P*-values. **C** Misclassification errors at each disturbance frequency level, via the leave-one-out allocation of observations to groups from CAP at each time point after d0 (*n* = 6 sampling days). Bray–Curtis β-diversity, squared root transformed data. Red diamonds display mean values. The box bounds the IQR divided by the median, and Tukey-style whiskers extend to a maximum of 1.5 times the IQR beyond the box. **D** Beta nearest taxon index (βNTI) at each disturbance frequency level, from pairwise comparisons across within-treatment replicates at each time point after d0 (*n* = 60 comparisons). Red diamonds display mean values. Notches show the 95% confidence interval for the median. When notches do not overlap, the medians can be judged to differ significantly. Shaded in gray is the zone where stochastic processes significantly dominate, |βNTI| < 2. βNTI values closer to zero indicate a higher relative contribution of stochastic assembly.

Community structure in terms of β-diversity showed temporal changes, which varied across disturbance levels for both Unifrac phylogenetic distances (Fig. 2A) and Bray–Curtis taxonomic distances (Fig. 2B). Unconstrained ordination displayed a dispersion effect in overall community structure over time, particularly after 7 days, with communities in each reactor diverting from each other (Fig. 2A). To disentangle the effect of disturbance from temporal dynamics in β-diversity, each time point was evaluated separately using constrained ordination via canonical analysis of principal coordinates (CAP) (Fig. 2B). Group-average cluster similarity (60%) was included to detect formations of clusters of community structure. Differences in β-diversity across disturbance levels were statistically significant at all time points evaluated (PERMANOVA *P*_adj_ < 0.001), without significant effects of heteroscedasticity (PERMDISP *P*_adj_ > 0.14) (Supplementary Table 1). Replicate reactors at the undisturbed (L0) and press disturbed level (L5) clustered separately from intermediate disturbance levels on almost all sampling days, except d7 and d21 for L0 (Fig. 2B), both levels having 0% misclassification error at all time points assessed (Fig. 2C). Comparatively, reactors at intermediate disturbance frequencies (L1-4) clustered together and showed higher dispersion across replicates within the same level, with CAP misclassification errors above zero (Fig. 2B, C). Thus, replicate reactors were less similar to each other at intermediate levels of disturbance, while replicates at low (undisturbed) and high (press disturbed) disturbance frequencies were more similar. Likewise, community assembly assessed via the beta nearest taxon index (βNTI)^42^ showed a higher relative contribution of stochasticity at intermediate levels of disturbance (Fig. 2D), with βΝΤΙ values closer to zero, indicating that phylogenetic turnover across within-treatment replicates was closer to the null expectation. Similarly to what we observed through the NTI, the relative importance of stochasticity decreased with time in the experiment (i.e., higher | βΝΤΙ| values) and when abundance weighing was included in the calculation of the βΝΤΙ values (βΝΤΙ_W_) (Supplementary Fig. 6). The observed temporal changes in bacterial community structure at the ASV level across disturbance frequencies were consistent with phylum- and genus-level dynamics of relative abundances (Supplementary Fig. 7), although the focus of this study was on overall community dynamics and not on any particular group of taxa.

### Community function dynamics and correlations with community structure and assembly

**Fig. 3:**
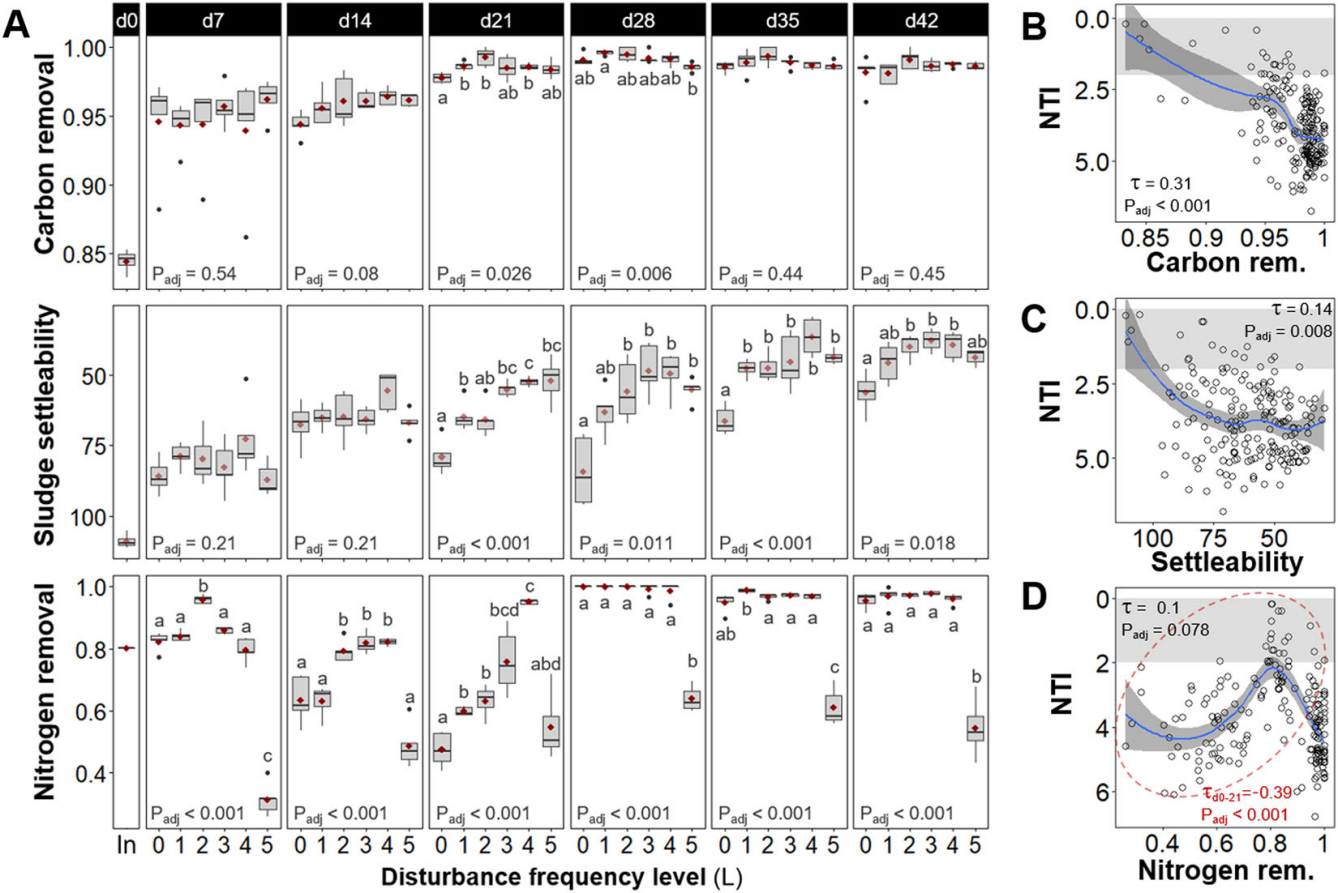
Community function dynamics. **A** Community function assessed via influent chemical oxygen demand removal (carbon removal as fraction of total, upper panels), sludge volume index (sludge settleability, middle panels), and influent total Kjeldahl nitrogen removal (nitrogen removal as fraction of total, lower panels) for different frequencies of organic loading disturbance (*n* = 5). Disturbance frequency levels (L): 0 (undisturbed), 1–4 (intermediately disturbed), 5 (press disturbed). In: sludge inoculum (day 0, *n* = 4). Each panel represents a sampling day, red diamonds display mean values. The box bounds the IQR divided by the median, and Tukey-style whiskers extend to a maximum of 1.5 times the IQR beyond the box. Characters above boxes display Games-Howell post hoc grouping (*P*_adj_ < 0.05). Welch’s ANOVA *P*-values adjusted at 5% FDR shown within panels. Correlations of **B** carbon removal, **C** sludge settleability, and **D** nitrogen removal, vs. NTI from bacterial ASV data across all frequency levels and time points evaluated in this study (*m* = 184). Kendall correlation *τ*- and adjusted *P*-values are indicated within the panels. Blue line represents locally estimated scatterplot smoothing regression (loess) with confidence interval in dark-gray shading. Shaded in gray is the zone of significant stochastic phylogenetic dispersion, |NTI | < 2. Red ellipse and *τ*- and *P*-value in panel **D** indicate data at initial stages of succession (d0 to d21). Note the inverted axis for sludge settleability, as it increases with decreasing SVI values, and for NTI, since values closer to zero indicate a higher relative contribution of stochastic assembly.

Bacterial community function was assessed over time via influent chemical oxygen demand (COD) removal, sludge volume index (SVI), and influent total Kjeldahl nitrogen (TKN) removal, as measure of carbon removal, sludge settleability and nitrogen removal, respectively (Fig. 3A). Carbon removal and sludge settleability, which are functions associated with a broad range of taxa (i.e., general functions), improved over time during the experiment. High-carbon removal (>0.97, fraction of total) was achieved at all disturbance frequency levels from d21 onwards, with no significant differences on days 35 and 42, after a period of high variability for same-level replicates during the first 14 days. Sludge settleability increased with disturbance frequency, with undisturbed (L0) reactors showing the lowest settleability from d21 onwards and intermediately disturbed levels reaching the highest settleability on d42 (SVI Welch’s ANOVA *P*_adj_ = 0.018). The nitrogen removal function (TKN removal), which is related to specialized bacteria (ammonia oxidizers), significantly differed across disturbance frequencies (TKN removal Welch’s ANOVA *P*_adj_ < 0.001) with the highest nitrogen removal at intermediately disturbed levels during the first 21 days. From d28 onwards, L0 to L4 reactors had similarly high average nitrogen removal (>0.9, fraction of total), and only the press disturbed reactors (L5) continued to have lower nitrogen removal (<0.7, fraction of total) than that of the initial sludge inoculum (0.8, fraction of total). Effluent values of TKN, ammonia, nitrite and nitrate showed that TKN removal occurred via nitrification (Supplementary Fig. 8).

Carbon removal had an overall significant positive Kendall correlation with α-diversity indices (*τ* = 0.21–0.25, *P*_adj_ < 0.001), whereas sludge settleability and nitrogen removal showed non-significant correlations with α-diversity across the study (Supplementary Fig. 9). Correlations between general functions of carbon removal and sludge settleability and both ΝΤΙ and ΝΤΙ_W_ were positive and significant across all time points and disturbance frequencies of the study (Fig. 3B, C and Supplementary Fig. 9), implying higher performance of these functions under stronger deterministic assembly mechanisms. Nitrogen removal had negative and significant correlations with ΝΤΙ and ΝΤΙ_W_ when only the first 21 days of the study were considered (ΝΤΙ *τ*_d0-21_ = −0.39, *P*_adj_ < 0.001, Fig. 3D; ΝΤΙ_W_ *τ*_d0-21_ = −0.46, *P*_adj_ < 0.001, Supplementary Fig. 10), suggesting a better performance of this function under higher stochasticity at intermediate disturbance frequencies during the first three weeks of the study.

## Discussion

**Fig. 4:**
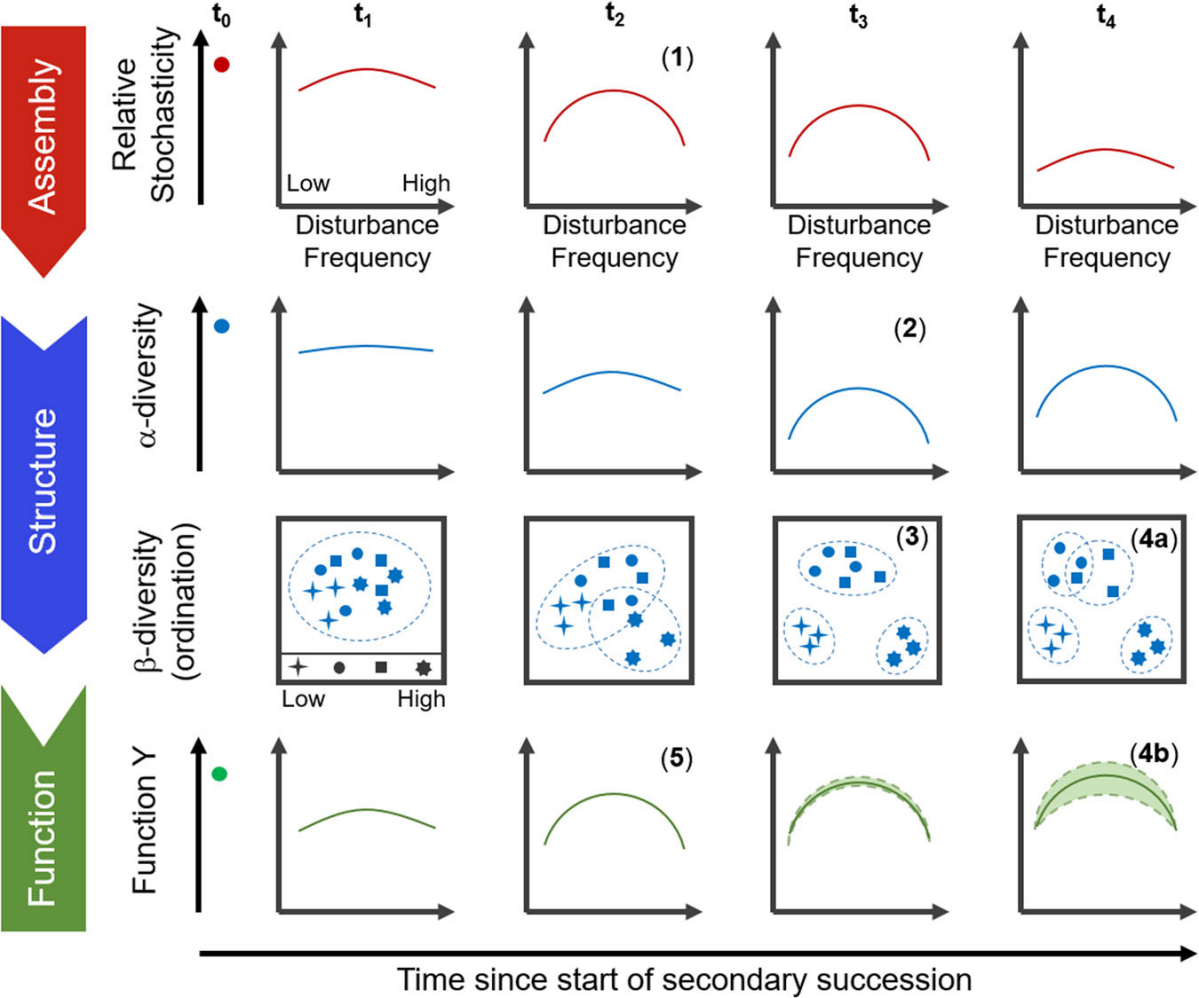
Conceptual representation of the intermediate stochasticity hypothesis (ISH) to describe patterns of assembly and structure along a disturbance frequency gradient, for communities in secondary succession (starting at time point *t*_0_). (**1**) Initially, stochastic assembly mechanisms (e.g., priority effects, historical contingency and legacy effects) are favored at intermediate disturbance frequencies, promoting re-colonization processes from the low-abundance fraction of the community or seed bank. (**2**) Subsequently, these are followed by changes in the community structure that manifest as a peak of α-diversity at intermediate levels of disturbance. (**3**) Over time, three separated clusters of β-diversity ordination may form comprising low, intermediate, and high levels of the disturbance range (in this schematic, *n* = 3). Furthermore, stochasticity operating at intermediate disturbance levels may lead to variable within-treatment (**4a**) β-diversity, visualized as partially overlapping cluster similarity (dashed ovals), and (**4b**) community function, displayed as wider confidence intervals (shaded area between dashed lines). (**5**) The output of some functions (in this study, nitrogen removal) could be higher at intermediate disturbance frequencies during initial successional stages due to higher stochastic assembly. Additionally, the overall relative contribution of stochasticity decreases with succession time (see top row of subpanels).

In this study, we found stochastic assembly processes to be more important at intermediate disturbance frequencies where the highest α-diversity was also observed, together with high β-diversity dispersion across within-treatment replicates as predicted by the ISH^9^. Furthermore, we showed that a peak in the relative contribution of stochasticity preceded a peak in α-diversity across a disturbance frequency range. These findings highlight the utility of the ISH to gain a mechanistic understanding of disturbance–diversity relationships by incorporating the role of assembly mechanisms (Fig. 4). Moreover, we observed that carbon removal and microbial aggregate settleability (general functions) correlated with higher deterministic processes, while higher stochasticity correlated with higher nitrogen removal (a specialized function) only during initial successional stages at intermediate disturbance frequencies. The function-assembly correlations observed in this study suggest that the ISH could also improve our understanding of disturbance–diversity–function relationships, but more research is needed to confirm this.

In our earlier work the presence of 3-chloroaniline had served as a disturbance^9^, since chlororanilines are toxic compounds known to inhibit nitrification and carbon removal in sludge bioreactors^43^, affecting both bacterial community structure and assembly^26^. Here, we expanded on it by doubling the organic loading as a different type of disturbance for a distinct microbial community inoculum, a relevant scenario given the multidimensional nature of disturbance^8^. Indeed, a doubling of organic loading was shown to have a negative effect on nitrification^37^, while also affecting the assembly and structure of bacterial communities in sludge bioreactors^27^. Further, the use of taxonomic and phylogenetic diversity metrics, in both unweighted and abundance-weighted forms, allowed us to cover a broader aspect of α-diversity. Taxonomic resolution was also improved by the use of amplicon sequence variants (ASVs) compared to operational taxonomic unit (OTU) clustering^44^ with about one to two orders of magnitude fewer spurious units^45^, allowing for a better estimation of unweighted α-diversity (i.e., taxa richness). We further verified that the observed patterns occurred independently of data rarefaction, given the lack of consensus about this practice^46^ and the fact that it is known to affect (mainly unweighted) estimations of α-diversity^47^. Assembly processes were tracked over time using a phylogenetic null modeling methodology, which has been used in various types of microbial community studies^25,42,48^. Additionally, general and specific functions were evaluated against structure and assembly. All these enhancements allowed us to test the ISH, while also gaining new insights into the role of assembly processes behind disturbance-induced changes in community structure and function over time.

Our experimental system produced a series of secondary succession scenarios in which bacterial communities had to adapt to various changes due to the transition from a full-scale system to a bioreactor microcosm configuration, along with different frequencies of disturbance. These changes included bioreactor type (continuous to batch), feed type (natural to synthetic wastewater), volume (full-scale to microcosm), cell residence time (low to high), and immigration (open to closed system), comparable to what we described in a prior study^49^. This shift was an initial severe disturbance and explains the initial temporal decrease in α-diversity at all disturbance frequency levels compared to the sludge inoculum. However, time of succession led to a significant hump-backed pattern of α-diversity for all composition- and abundance-based indices employed in the study, which occurred after 21 days for ^2^D, 28 days for ^1^D, PD and PD_W_, and 35 days for ^0^D. Thus, the observed dynamics in community structure were stronger in terms of relative abundances than richness (^2^D, ^1^D vs. ^0^D), as well as at the phylogenetic vs. taxonomic level (PD vs. ^0^D). The appearance of higher phylogenetic α-diversity at intermediate levels of disturbance for both unweighted (PD) and abundance-weighed (PD_W_) indices suggests that considering evolutionary relationships among organisms^50^ could also aid in assessing the effect of varying disturbances on community structure under succession. In our study, disturbance promoted the co-occurrence of organisms with large phylogenetic distances, suggesting that additional niches were created at intermediate disturbance frequencies that were occupied by ecologically different species, thus reducing competitive exclusion. Conversely, phylogenetic clustering at undisturbed and press disturbed levels can be interpreted as communities structured by environmental filtering^41^. Additionally, temporal analysis of community structure in terms of β-diversity revealed three different clusters for undisturbed, press disturbed and intermediately disturbed reactors. Further comparison of replicates within the same disturbance frequency level showed higher β-diversity variability at intermediate disturbance levels, which was coherent with prior observations in freshwater ponds^51^ and sludge bioreactors^9^ where β-diversity increased with stochastic assembly. Our findings are relevant for understanding disturbance–diversity relationships, since few studies have reported parabolic α-diversity patterns using abundance-based indices^12^. Furthermore, variations in β-diversity among ecological communities that are subject to large and fluctuating disturbances are believed to provide insights about the mechanisms driving changes in α-diversity and function^52^.

We observed similar trends of phylogenetic dispersion within a single community (NTI) and the phylogenetic turnover between communities of the same treatment level (βNTI), compared to the null expectation. Stochasticity was more important during initial successional stages of the study, with initial NTI and βNTI values closer to zero (i.e., closer to the null expectation of the model). Relatively, the overall strength of deterministic processes increased with time, with higher |NTI | and | βNTI | values. Similarly, late succession stages were shown to be governed by deterministic processes in plant forest^53^ and microbial groundwater communities^54^. Furthermore, α-diversity-based temporal assembly dynamics revealed a parabolic pattern in ΝΤΙ and ΝΤΙ_W_, through the disturbance frequency gradient, which was evident after 14 and 7 days of the study, respectively, before the appearance of similar parabolic patterns across various α-diversity indices. This preceding pattern is considered here as a strong indicator of assembly mechanisms operating to shape community structure. It is, therefore, plausible that stochastic assembly mechanisms were first favored at intermediate disturbance frequencies, prompting subsequent changes of community structure that resulted in the observed higher α-diversity as the ISH proposes^9^.

Our observations are also coherent with the idea that secondary succession is community assembly in action^55^. The disturbance range in this study produced different secondary succession scenarios, with communities in the sludge of each bioreactor likely experiencing different re-colonization processes from their bacterial seed bank (i.e., low-abundance or rare taxa), via stochastic processes such as priority effects^29^ followed by historical contingency^56^ and legacy effects^3^. Importantly, external dispersal processes^57^ (i.e., bacterial immigration) could not influence community assembly since bioreactors in this study were operated as closed systems. Indeed, microbial seed banks are thought to contribute to the maintenance of microbial diversity^58^ and have been described as essential for understanding temporal community changes^59^. Further, stochastic assembly processes were shown to be more preponderant within the rare fraction of the microbial community^27^. Nonetheless, other processes might also be promoting stochastic assembly at intermediate disturbance frequencies, like ecological drift^48^ and feedback mechanisms linked to density dependence and species interactions^60^. Hence, a disturbance frequency gradient can not only result in nonlinearities for growth rates that would affect the outcome of competition^8,18^, it could also alter the relative contribution of stochastic and deterministic mechanisms of community assembly that underlie changes in community structure^9^. Furthermore, our results showed that, over a range of disturbance frequencies, assessing temporal community assembly patterns during succession can act as a sentinel of upcoming patterns of diversity.

Stochasticity was positively correlated with better nitrogen (as TKN) removal via nitrification at intermediate disturbance frequencies during the initial successional stages where stochastic processes were also generally prevalent. Nitrification functions are carried out by specific taxa (i.e., nitrifiers), which are slow growers, nutritionally inflexible, sensitive to inhibitors and less phylogenetically diverse than many other key functional guilds^61^. Yet, the recruitment of nitrifying organisms from the microbial seed bank was important for the recovery of nitrification, following the removal of a long-term disturbance of altering food-to-biomass and carbon-to-nitrogen ratios in sludge bioreactors, although resilience varied across identically treated replicates^37^. Also, partial recovery of nitrification in sludge bioreactors was observed at intermediate frequencies of 3-chloroaniline disturbance, where stochastic assembly processes and within-treatment variability were also higher^9^. Conversely, general functions of carbon removal and settleability performed better when deterministic processes were stronger (higher | NTI | values). Carbon removal was better when α-diversity was lower, similarly to what was reported previously using a different xenobiotic disturbance in bioreactors^9^. Hence, a more diverse community does not necessarily translate into better ecosystem functions^9,62^. Our data suggest that general functions thrive during stronger deterministic processes, while specialized functions might be favored by stochasticity at initial successional stages. A limitation of this study was the use of only one specialized and two general functions. Future studies assessing the effect of fluctuating disturbances on community diversity and function should also consider the type of function (e.g., specific or general), the stage of succession after the disturbance, and the underlying assembly mechanisms.

The observed patterns in community assembly, structure and function were time dependent. The ISH successional pattern appears to be transient, as assembly mechanisms across disturbance frequency levels were not significantly different towards the end of the study on d42, while α-diversity continued to display a significant parabolic pattern. If the gradient of disturbance frequencies is maintained over time, then the peak in α-diversity at intermediate levels might continue during the late successional stages, but this remains to be investigated. Nonetheless, most relevant bacteria in activated sludge have generation times of less than 24 h. Hence, the 42-day length of this study represented around tens to hundreds of generations of many different taxa, allowing the detection of significant patterns in assembly and structure.

Further research in a variety of ecosystems is needed to validate the broad applicability of the ISH, particularly considering that disturbance can vary in type, frequency, intensity, driver and impact^8,34^. A diversity disturbance relationship can be affected by the interaction between disturbance frequency and intensity^8^. This was shown in microcosm studies involving genetically distinct morphotypes of *Pseudomonas fluorescens* cultures^63^, freshwater bacterial communities^64^, and soil-derived bacterial communities in chemostats^65^, using biomass removal^63,64^ and dilution^64,65^ as disturbances. Although none of these studies quantified community assembly mechanisms, a higher variability of bacterial community structure at intermediate disturbance treatments was reported for the case of freshwater microcosms^64^, suggesting that this could be related to a transition to stochastic community assembly, in accordance with the ISH. For activated sludge microcosms, the effect of different intensities (i.e., concentrations) of 3-chloroaniline^9^ or double organic loading addition (this study) to the bioreactors under a gradient of feeding frequencies remains to be investigated. Moreover, the definition of a given disturbance range is arbitrary, yet non-trivial^20^, and could obscure the pattern predicted by the ISH.

Studies on different scales are also necessary since ecological patterns can vary across spatial, temporal and phylogenetic scales^3^, while the effect of dispersal processes could also be evaluated within open systems. Although a similar study on communities of larger organisms would require considerably larger scales of space and time, some modeling approaches suggest that ISH-like patterns (Fig. 4) could emerge in community assembly and structure under varying disturbances. For example, forest fire modeling showed that intermediate lightning strike frequencies yielded higher species diversity, but also resulted in a greater role for stochasticity in driving the system on diverse trajectories^66^. Likewise, a conceptual model developed for plants and animals suggested that high variation in resource abundance and location in space and time, which could be caused by disturbance, would favor diversity via adaptation through novelty and innovation (i.e., stochasticity) generation^67^.

The predictions of the ISH could help to identify cases when disturbance-induced stochastic assembly promotes alternative states of community structure that compromise or enhance ecosystem function, to design mitigation or intensification strategies. Furthermore, it could be used to promote community resistance and resilience to future disturbances via increased α-diversity and functional-gene diversity. Alternatively, this theoretical framework could help in the design of functionally resilient communities that do not occur naturally, through the stochastic mechanisms that are initially elicited at intermediate frequencies of disturbance and provide an advantage to rare or low-abundance taxa. Therefore, we posit that the ISH may provide a general understanding of disturbance-induced changes in community structure and function during succession, by integrating the influence of the underlying assembly processes over time.

## Materials and methods

### Experimental design and function analyses

We employed 30 sequencing batch bioreactors at a microcosm scale (25-mL working volume), inoculated with activated sludge from a full-scale wastewater treatment plant in Singapore and operated for 42 days at 30 °C in an incubator shaker. The daily complex synthetic feeding regime included double organic loading at varying disturbance frequencies. Six levels of disturbance were set in quintuplicate independent reactors (*n* = 5), which received double organic loading either never (undisturbed), every 8, 6, 4, or 2 days (intermediately disturbed), or every day (press disturbed) (Supplementary Fig. 11). Level numbers were assigned from 0 to 5 (0 for no disturbance, 1 to 5 for low to high disturbance frequency). Disturbance frequency was further calculated from the rate of high organic loading at each disturbance level resulting in values of 0,^1^/_8_,^1^/_6_,^1^/_4_,^1^/_2_, and 1. The number of double organic loading events at each disturbance frequency level (i.e., disturbance incidence) during the 42 days of the study was 0, 6, 8, 11, 22, and 42 (Supplementary Fig. 11). Ecosystem function, in the form of process performance parameters at the end of a cycle, was measured weekly in accordance with Standard Methods^68^ where appropriate, and targeted the removal of soluble COD and TKN from the mixed liquor after feeding. COD (Standard Methods 5220 D) and nitrogen species (ammonium, nitrite, and nitrate ions) were measured using spectrophotometric tests (Hach) and ion chromatography (Standard Methods 4500-NH3 for ammonium; 4110 B for nitrate and nitrite). The COD measured was adjusted by subtracting the contribution of nitrite on the basis of 1.1 g COD/g NO_2_^−^-N to correct for nitrite interference. Total nitrogen (TN) was measured using a TOC-L analyser (Shimadzu) and used to estimate TKN content (TKN = TN – NO_2_^−^-N - NO_3_^−^-N). Effluent samples were filtered through a 0.2-μm pore size filter and the filtrate was stored at 4 °C for less than 1 week prior to chemical analyses. Sludge settling capacity was measured via the SVI (mL/g), considering 30 min of settling time. Concentrations in the mixed liquor of the bioreactors after feeding (i.e., beginning of a new cycle) were regularly 305.8 (±7.4) mg COD/L and 45.6 (±0.8) mg TKN/L, or 594.7 (±18.6) mg COD/L and 46.1 (±0.2) mg TKN/L when double organic loading occurred. A food-to-biomass ratio (F:M) control approach was used as in Santillan et al.^37^, for which biomass was measured weekly as total suspended solids (TSS) after which sludge wastage was done to target a TSS of 1500 mg/L. The latter resulted in average solids residence time (SRT) values of 30, 26, 23, 22, 19, and 15 d, for disturbance levels from 0 to 5, respectively. Note that these SRT values are well above the doubling times of relevant bacteria in activated sludge^69^.

### Sludge inoculum collection and experiment setup

Sludge inoculum was collected from one of the activated sludge tanks of a water reclamation plant in Singapore, with a Modified Ludzack-Ettinger (MLE) process configuration. Operation parameters were: *Q* ≈ 200,000 m^3^/day, *T* ≈ 30 °C, pH ≈ 6.7, total suspended solids (TSS) ≈ 1500 mg/L, hydraulic residence time (HRT) ≈ 8–12 h, and solids residence time (SRT) ≈ 5–6 day. Typical influent concentrations were: total Kjeldahl nitrogen (TKN) ≈ 49 mg/L and total chemical oxygen demand (COD) ≈ 320 mg/L. The plant receives a mix of residential, commercial and industrial wastewater as its influent, operating continuously at C:N ≈ 6.5 mg COD/mg TKN and F:M ≈ 0.2–0.3 mg COD/mgTSS/day. It had a removal efficiency of around 80% for N and 90% for COD. On the day the sludge inoculum was collected, the average (±s.d.m. for *n* = 4) soluble influent concentrations to the secondary treatment were (in units of mg/L): COD = 220.7 ± 2.9, NH_4_^+^-N = 37.4 ± 0.8, TKN = 44.6 ± 0.4, NO_2_^−^-N = 0.00 ± 0.00, NO_3_^−^-N = 0.03 ± 0.00, PO_4_^3−^-P = 4.81 ± 0.02. Likewise, the soluble effluent concentrations from the secondary treatment were (in units of mg/L): COD = 34.3 ± 2.1, NH_4_^+^-N = 6.4 ± 0.1, TKN = 8.8 ± 0.1, NO_2_^−^-N = 0.01 ± 0.00, NO_3_^−^-N = 0.03 ± 0.00, PO_4_^3−^-P = 0.25 ± 0.04. With these values we estimated an influent COD removal of 0.84 ± 0.01 and an influent TKN removal of 0.80 ± 0.00. Activated sludge was collected in a 20-L container and immediately transported to the lab. The SVI of the inoculum sludge was 108.9 ± 2.2 mL/g, considering 30 min of settling time. The suspension was manually mixed by shaking the closed container thoroughly before transferring half of it to a 10-L vessel that was stirred using a magnetic stir plate to ensure homogeneity. Samples of 25 mL were transferred to thirty 50-mL tubes (Eppendorf), which served as sequencing batch reactors (SBR) in a microcosm setup. Tubes were numbered from 1 to 30 and the experimental units were randomly assigned. About 30 min of settling time was allowed and 12.5 mL of supernatant was removed and replaced with 12.5 mL of synthetic wastewater with or without double organic loading as described below. On the first day a mix of synthetic wastewater with double organic loading was added to reactors for levels 1 to 5, while level 0 reactors received regular synthetic wastewater. All reactors were capped and incubated until the following day in an incubator shaker at 30 °C, the prevailing water temperature for wastewater treatment plants in Singapore. After each cycle (24 h) all the tubes were removed from the incubator and allowed to settle for 30 min, after which 12.5 mL of “effluent” supernatant liquid was removed and replaced aseptically with 12.5 mL of fresh synthetic medium, resulting in a 48-h HRT.

### Bioreactor feeding and complex synthetic wastewater preparation

The composition of the regular synthetic wastewater in the bioreactor feed was adapted from Santillan et al.^37^. It contained the following compounds, expressed in mg/L in mixed liquor in each reactor right after feeding: yeast extract (19.8), soy peptone (18.4), meat peptone (26.3), casein peptone (27.7), sodium acetate anhydrous (119.9), dextrose anhydrous (95.9), urea (37.7), ammonium bicarbonate (33.9), ammonium chloride (63.7), sodium dihydrogen phosphate monohydrate (27.3), sodium phosphate dibasic dihydrate (4.9), calcium chloride dihydrate (50.0), magnesium sulfate heptahydrate (112.5), and sodium bicarbonate (180), which was added to replace the alkalinity consumed during nitrification. The medium also contained 0.25 mL/L of a trace element stock, which contained (g/L) citric acid monohydrate (5), EDTA acid disodium salt dihydrate (1.2), hippuric acid (4), sodium molybdate dihydrate (0.24), potassium iodide (0.24), sodium tungstate dihydrate (0.24), boric acid (1), cobalt(II) chloride hexahydrate (0.24), copper(II) sulfate pentahydrate (0.48), manganese(II) chloride tetrahydrate (0.96), nickel(II) chloride hexahydrate (0.24), nitrilotriacetic acid trisodium salt monohydrate (2.88), iron(III) chloride hexahydrate (12), zinc sulfate heptahydrate (1.2).

The above values were used to prepare batches of 2-L bottles of sterile media, a total of six to be used as regular bioreactor feed and three for double organic loading feeding. Average feed concentrations of 305.8 (±7.4) mg COD/L and 45.6 (±0.8) mg TKN/L in the mixed liquor after feeding (i.e., beginning of a new cycle) for reactors. Reactors under double organic loading received double the amount of yeast extract (39.5), soy peptone (36.9), meat peptone (52.7), casein peptone (55.3), sodium acetate anhydrous (239.7), and dextrose anhydrous (191.8), as well as less urea (27.5), ammonium bicarbonate (24.7), and ammonium chloride (46.5) to compensate for the increase in organic TKN. This resulted in average feed concentrations of 594.7 (±18.6) mg COD/L and 46.1 (±0.2) mg TKN/L in the mixed liquor after feeding, when applying double organic loading. Phosphate addition targeted a concentration in mixed liquor of 7.45 (±0.8) mg P/L to obtain a N:P of around 6. The synthetic medium to be used for the whole study was prepared on the same day and filtered through a 0.2-μm pore size filter to avoid contamination. The filtrate was stored at 4 °C for the duration of the study and handled in aseptic conditions.

### DNA extractions

Sludge samples of 2 mL were collected on the initial day of the study (four samples, taken at random from the inoculum mix) and weekly from each reactor afterwards (180 samples), and stored at −80 °C for DNA extraction. Genomic DNA was extracted from about 500 μL of sludge using the FastDNA Spin Kit for Soil and the FastPrep Instrument (MP Biomedicals) with modifications to the manufacturer’s protocol to increase DNA yield^49^. The first modification involved performing four lysis cycles in the FastPrep instrument instead of one, with two min of rest in between each cycle, during which the samples were placed on ice. The second modification involved eluting DNA from the spin column using nuclease-free water that had been pre-heated to 55 °C, followed by incubation of the columns in elution water at 55 °C for 5 min before the final centrifugation. Extracted DNA was quantified using both NanoDrop 2000c and Qubit 3.0 fluorometer (both ThermoFisher Scientific), and purified using the Genomic DNA Clean & Concentrator kit (Zymo Research) following the protocol from the manufacturer.

### 16S rRNA gene metabarcoding and reads processing

Bacterial 16S rRNA metabarcoding was done in two steps^37^. For the first PCR stage, each reaction (25 μL) contained 12.5 μL of HiFi Hotstart Readymix (Kapa Biosystems), 9.5 μL of nuclease-free water, 0.5 μL (each) of forward and reverse primers (10 μM) and 2 μL of DNA template (6 ng/μL). Primer set 341f/785r targeted the V3-V4 variable regions of the 16S rRNA gene^70^. Thermocycler settings were: Initial denaturation at 95 °C for 2 min, 30 cycles of 95 °C for 30 s, 58 °C for 15 s, 72 °C for 30 s, and final elongation at 72 °C for 2 min. PCR reactions were all run in duplicate and pooled subsequently. Amplicon libraries were purified using the Agencourt AMpure XP bead protocol (Beckmann Coulter). Library concentration was measured with Qubit 3.0 fluorometer (Thermo Fisher Scientific) and quality validated with a Tapestation 2200 (Agilent). The second stage PCR (Indexing PCR) was performed according to the recommendations in Illumina’s ‘16S Metagenomic Sequencing Library Preparation’ application note. This step uses a limited 8-cycle PCR to complete the Illumina sequencing adapters and add dual-index barcodes to the amplicon target. Five microliters of the intermediate PCR product from the first stage were used as template for the indexing PCR and samples were amplified with 8 PCR cycles. Nextera XT v2 indices were used for dual-index barcoding to allow pooling of the amplicon targets for sequencing. Finished amplicon libraries were quantitated using QuantiFluor dsDNA assay (Promega) and the average library size was determined on a Tapestation 4200 (Agilent). Library concentrations were then normalized to 4 nM and validated by qPCR on a QuantStudio-3 system (Applied Biosystems), using the Kapa library quantification kit for Illumina platforms (Kapa Biosystems). The libraries were then pooled at equimolar concentrations and sequenced on an Illumina MiSeq platform (v.3) with 20% PhiX spike-in and at a read-length of 300 bp paired-end read-length. Sequencing was done in-house at SCELSE’s core sequencing facility.

Sequenced sample libraries were processed with the *dada2* (v.1.3.3) R-package^45^, allowing inference of ASVs^44^. Illumina adaptors and PCR primers were trimmed prior to quality filtering. Sequences were truncated after 280 and 255 nucleotides for forward and reverse reads, respectively. After truncation, reads with expected error rates higher than 3 and 5 for forward and reverse reads, respectively, were removed. After filtering, error rate learning, ASV inference and denoising, reads were merged with a minimum overlap of 20 bp. Chimeric sequences (0.17% on average) were identified and removed. For a total of 184 samples, an average of 18,086 reads were kept per sample after processing, representing 47% of the average forward input reads. Taxonomy was assigned using the SILVA database (v.132)^71^. Diversity and assembly analyses were carried on both unrarefied and rarefied datasets. To generate the rarefied dataset, samples were rarefied to the lowest number of reads (5089) in a sample after processing (Supplementary Fig. 12).

### Bacterial community structure analyses and statistics

All reported *P*-values for statistical tests in this study were corrected for multiple comparisons using a false-discovery rate (FDR) of 5%. Hill diversity indices^39^ were used to quantify taxonomic α-diversity as described elsewhere^9^. Phylogenetic α-diversity was assessed through Faith’s phylogenetic distance^40^ (PD) including its abundance-weighted version (PD_W_). Community structure in terms of taxonomic β-diversity was evaluated through: (i) canonical analysis of principal coordinates (CAP) ordination including ellipses of 60% group-average cluster similarity; (ii) misclassification error analysis for each disturbance frequency level over the six time points sampled, via the leave-one-out allocation of observations to groups from CAP; and (iii) multivariate tests of permutational analysis of variance (PERMANOVA) and permutational analysis of dispersion (PERMDISP); all from Bray–Curtis dissimilarity matrixes at each time point sampled (30 bioreactors, *n* = 5), constructed from square-root transformed abundance data using PRIMER (v.7)^72^. Phylogenetic β-diversity was assessed via non-metric multidimensional (NMDS) ordination of a weighted Unifrac distance matrix, constructed from Hellinger transformed abundance data of all 184 samples using the *phyloseq*^73^ R-package (v.1.30.0) in R. The *ggplot2* package (v.3.3.2) in R^74^ was used for local polynomial regression fitting via the *loess* function (including 95% confidence intervals) and box plots construction (using Tukey-style whiskers). The *ggdist* R-package (v.2.4.1) was used to make the βNTI raincloud plot. Univariate testing through Welch’s analysis of variance (ANOVA) with Games-Howell post hoc grouping was done using the *rstatix*^75^ (v.0.6.0) R-package. Kendall correlations were done using the *ggpubr*^76^ package (v.0.4.0) in R. Heat maps for bacterial phyla relative abundances were constructed using the *ampvis2*^77^ package (v.2.6.2) in R.

### Bacterial community assembly analyses and statistics

The effect of underlying assembly mechanisms was assessed using phylogenetic-based null modeling approaches on both α- and β-diversity. First, the nearest taxon index (NTI)^41^ was calculated for each community to assess whether α-diversity was more or less structured than would be expected by random chance. The model uses the mean nearest taxon distance (MNTD)^41^, which quantifies the phylogenetic distance between each ASV in one community, as a measure of the clustering of closely related ASVs. Phylogenetic relatedness of ASVs was characterized by multiple-alignment of ASV sequences using *decipher* (v.2.14.0) R-package^78^. The phylogenetic tree was then constructed and a GTR + G + I maximum likelihood tree was fitted using the *phangorn* (v.2.5.5) R-package^79^. To quantify the degree to which MNTD deviates from a null model expectation, ASVs and abundances were shuffled across the tips of the phylogenetic tree. After shuffling, MNTD was recalculated to obtain a null value, and repeating the shuffling 1000 times provided a null distribution. Then, NTI was calculated as the difference between the mean of the null distribution and the observed MNTD in units of standard deviation^41^. The closer to zero a NTI value is, the closer to the null expectation (i.e., higher stochasticity) is the phylogenetic dispersion of a given community. Positive NTI values suggest phylogenetic clustering while negative values indicate phylogenetic overdispersion. Second, β-diversity null modeling via the β-nearest taxon index (βNTI) was done to investigate if the phylogenetic turnover across two samples was significantly more or less similar than would be expected by just random chance^42^. The model uses the β-mean nearest taxon distance (βMNTD), which quantifies the phylogenetic distance between pairs of ASVs drawn from two distinct communities. To quantify the degree to which βMNTD deviates from a null model expectation, ASVs and abundances were shuffled across the tips of the phylogenetic tree. After shuffling, βMNTD was recalculated to obtain a null value, and repeating the shuffling 1,000 times provided a null distribution. Then, βNTI was calculated as the difference between the mean of the null distribution and the observed βMNTD in units of standard deviation^42^. The closer to zero a βNTI value is, the closer to the null expectation (i.e., higher stochasticity) is the phylogenetic turnover between two communities. By convention, a value of | βNTI | > 2 indicates that the observed turnover is significantly deterministic, while | βNTI | < 2 indicates dominance of stochastic assembly processes^25^. Similarly, here we consider that |NTI | < 2 indicates dominance of stochastic phylogenetic clustering. Both unweighted and abundance-weighted NTI and βNTI values were calculated. These analyses were done using the *metagMisc*^80^ (v.0.0.4) and *picante*^81^ (v.1.8.2) R-packages. To test for a phylogenetic signal across phylogenetic distances, Mantel correlograms were constructed using the *vegan*^82^ (v.2.5.6) R-package, relating between-ASV niche differences to between-ASV phylogenetic distances across a given phylogenetic distance, following the methodology by Dini-Andreote et al.^25^. Environmental niches were constructed from bioreactor effluent process data (COD removal, TKN removal and SVI). Values for each bioreactor effluent process variable were normalized as standard normal deviates. For each ASV we calculated its relative-abundance-weighted mean value for each bioreactor effluent process variable. The resulting values estimate the magnitude of each bioreactor effluent process variable at which a given ASV is most abundant, which is interpreted as a proxy for the level of each bioreactor effluent process variable at which a given ASV has its highest fitness^25,42^ (i.e., the ASV’s environmental niche with respect to a given bioreactor effluent process variable). After estimating environmental niches for all ASVs with respect to all bioreactor effluent process variables considered, a matrix containing these estimates was generated with ASVs as rows and bioreactor effluent process variables as columns. Subsequently, among-ASV differences in environmental optima were then quantified as Euclidean distances simultaneously using all bioreactor effluent process axes. Thus, the Mantel correlogram involved two distance matrices having ASVs as both rows and columns, one matrix containing between-ASV differences in environmental niches, and the other matrix containing between-ASV phylogenetic distances. Phylogenetic distances were then quantified for 50 phylogenetic distance bins, while the significance of Pearson correlations was assessed using 1000 permutations and FDR (5%) correction.

### Reporting summary

Further information on research design is available in the Nature Research Reporting Summary linked to this article.

## Supplementary information

Supplementary Information

Supplementary Dataset 1

Supplementary Dataset 2

NP Reporting Summary

## Data Availability

DNA sequencing data are available at NCBI BioProjects with accession number PRJNA723443. See Supplementary Information for additional figures of diversity and community assembly metrics, correlations, heat maps and data rarefaction. Diversity analyses on rarefied data and all other relevant data to reproduce the results of this study are available as supplementary files in the online version of this manuscript.
