# Supplementary material for: Microbiome assembly predictably shapes diversity across a range of disturbance frequencies in experimental microcosms
Source: NPJ Biofilms Microbiomes. 2022 May 13;8:41. doi: 10.1038/s41522-022-00301-3 (PMC9106739; doi:10.1038/s41522-022-00301-3)
Supplement: Supplementary file 1 — Supplementary Information [file 41522_2022_301_MOESM1_ESM.pdf]

Supplementary Information

Microbiome assembly predictably shapes diversity across a range of disturbance frequencies in experimental microcosms

Authors: Ezequiel Santillan[1] and Stefan Wuertz[1,2]*

Affiliations:

[1]Singapore Centre for Environmental Life Sciences Engineering, Nanyang Technological University, 637551, Singapore.

[2]School of Civil and Environmental Engineering, Nanyang Technological University, 639798, Singapore.

*Correspondence to: Stefan Wuertz, swuertz@ntu.edu.sg

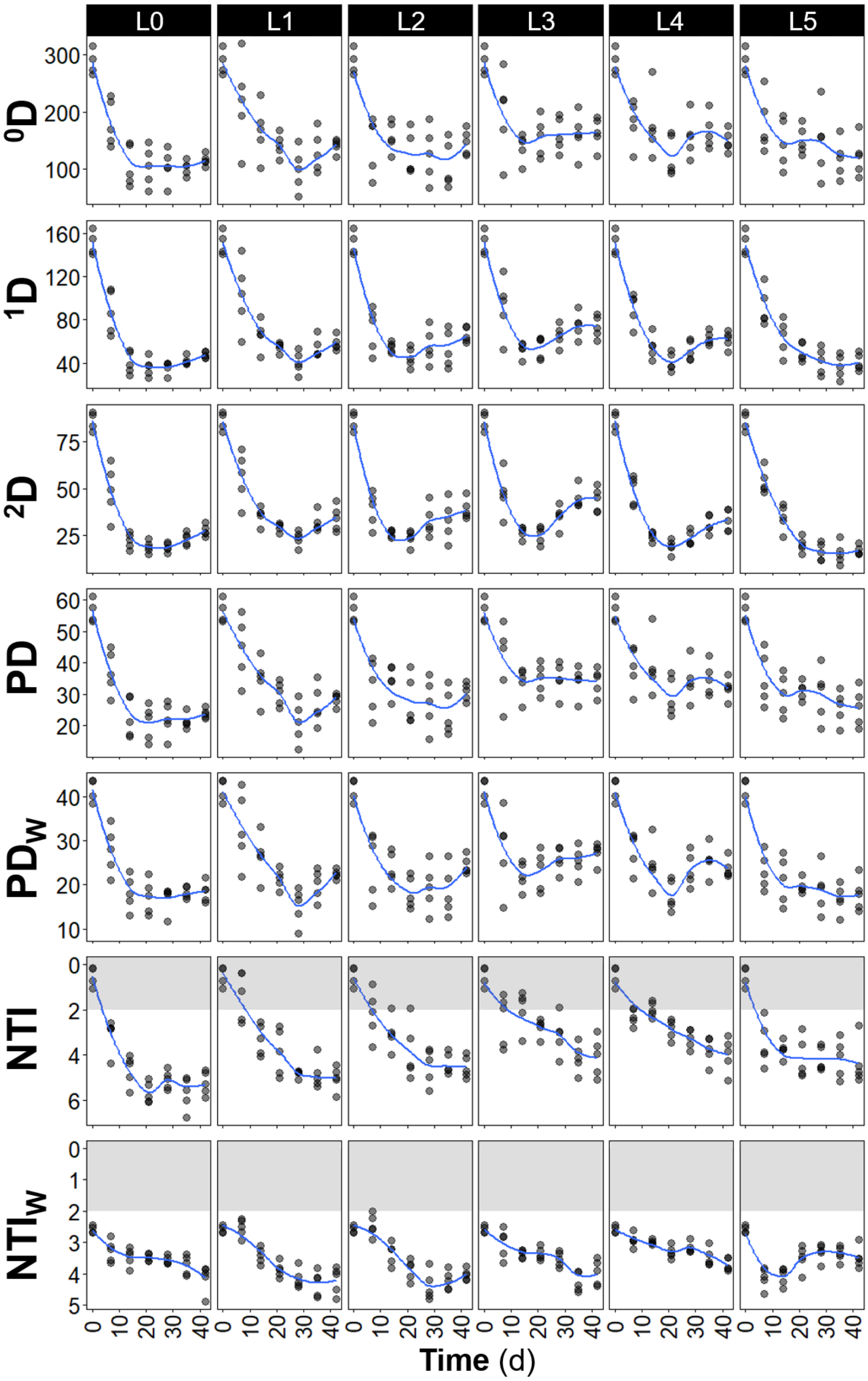

Supplementary Figure 1 – Temporal dynamics of richness (^{0}D), Hill α-diversity of 1st (^{1}D) and 2nd (^{2}D) order, phylogenetic diversity unweighted (PD) and abundance-weighted (PD_W), nearest taxon index unweighted (NTI) and abundance-weighted (NTI_W), from bacterial ASV data for each frequency of organic loading disturbance (n = 5, except for day 0 where n = 4). Disturbance frequency levels (L): 0 (undisturbed), 1-4 (intermediately disturbed), 5 (press disturbed). Blue line represents locally estimated scatterplot smoothing regression (loess). Note the inverted y-axis for both NTI and NTI_W, as values closer to zero indicate a higher relative contribution of stochastic assembly. Shaded in grey is the zone of significant stochastic phylogenetic dispersion, $|NTI| < 2$ and $|NTI_W| < 2$.

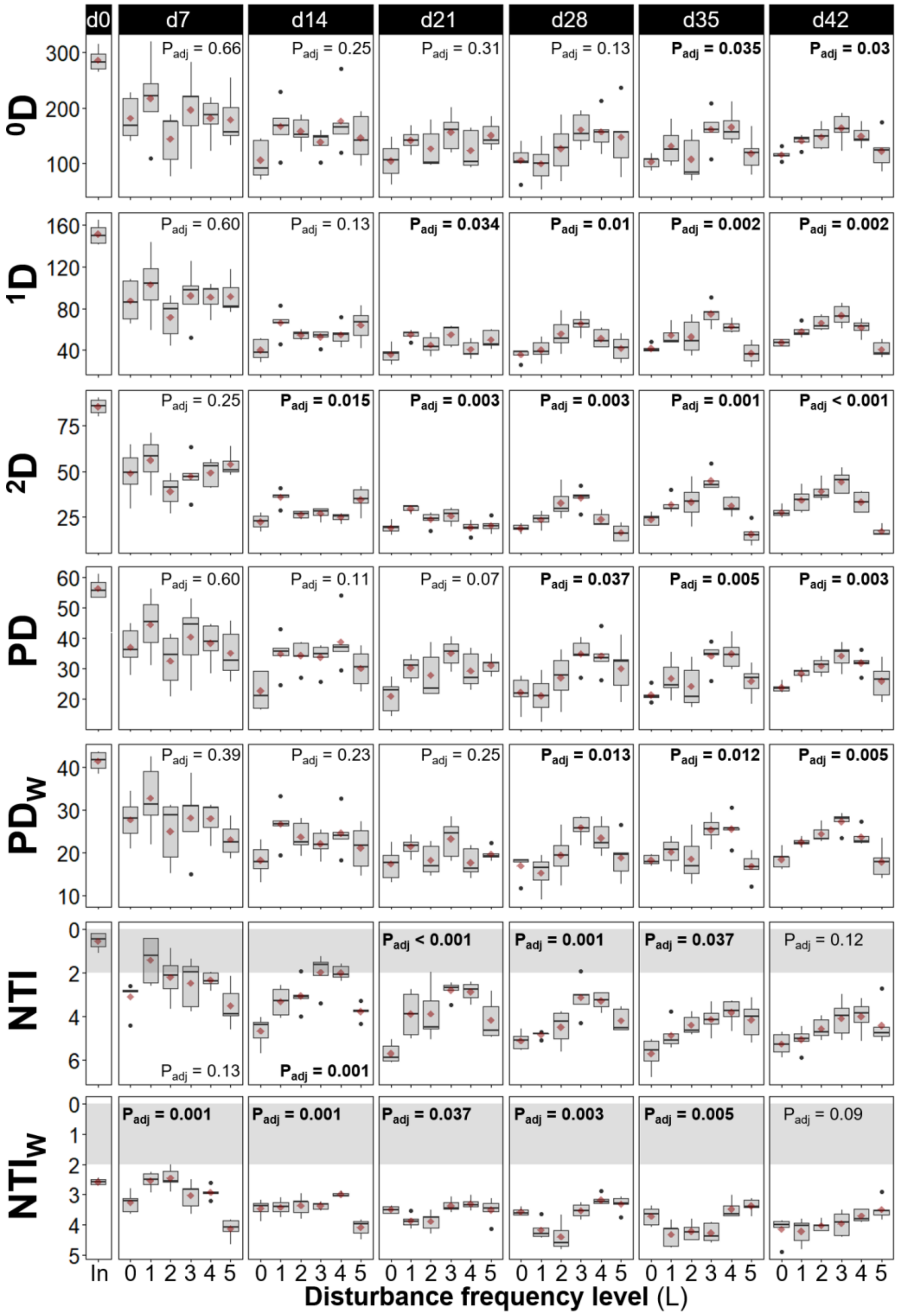

Supplementary Figure 2 – Community structure and assembly assessed via richness (^{0}D), Hill α-diversity of 1st (^{1}D) and 2nd (^{2}D) order, phylogenetic diversity unweighted (PD) and abundance-weighted (PD_W), nearest taxon index unweighted (NTI) and abundance-weighted (NTI_W), from bacterial ASV data for different frequencies of organic loading disturbance (n = 5). Disturbance frequency levels (L): 0 (undisturbed), 1-4 (intermediately disturbed), 5 (press disturbed). In: sludge inoculum (day 0, n = 4). Each panel represents a sampling day, red diamonds display mean values. Welch's ANOVA P-values adjusted at 5% FDR shown within panels. Note the inverted y-axis for both NTI and NTI_W, as values closer to zero indicate a higher relative contribution of stochastic assembly. Shaded in grey is the zone of significant stochastic phylogenetic dispersion, $|NTI| < 2$ and $|NTI_W| < 2$. The box bounds the interquartile range (IQR) divided by the median, and Tukey-style whiskers extend to a maximum of 1.5 times the IQR beyond the box.

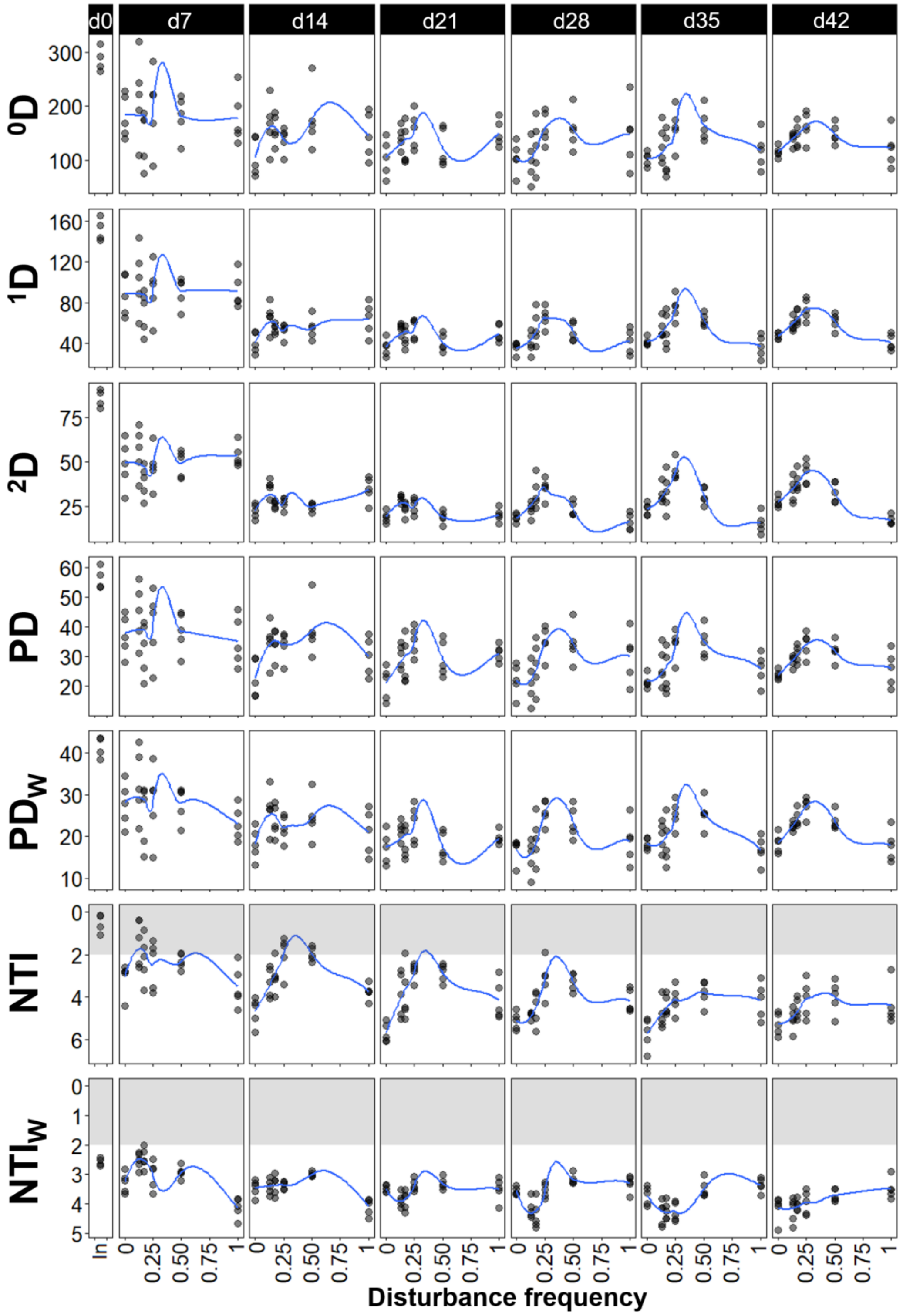

Supplementary Figure 3 – Community structure and assembly assessed via richness (^{0}D), Hill α-diversity of 1st (^{1}D) and 2nd (^{2}D) order, phylogenetic diversity non-weighed (PD) and abundance-weighed (PD_W), nearest taxon index unweighted (NTI) and abundance-weighted (NTI_W), from bacterial ASV data for different frequencies of organic loading disturbance (n = 5). Disturbance frequency values were calculated from the frequency of the high organic loading at each disturbance level. In: sludge inoculum (day 0, n = 4). Each panel represents a sampling day. Blue line represents locally estimated scatterplot smoothing regression (loess). Note the inverted y-axis for both NTI and NTI_W, as values closer to zero indicate a higher relative contribution of stochastic assembly. Shaded in grey is the zone of significant stochastic phylogenetic dispersion, $|NTI| < 2$ and $|NTI_W| < 2$.

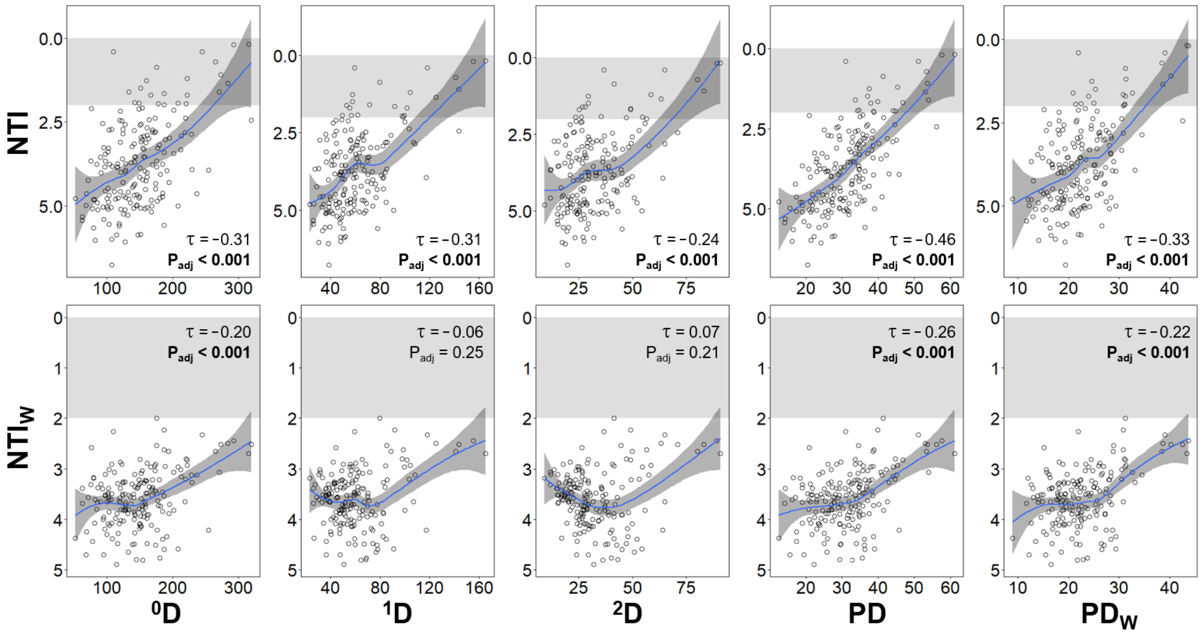

Supplementary Figure 4 – Richness (^{0}D), Hill α-diversity of 1st (^{1}D) and 2nd (^{2}D) order, unweighted (PD) and abundance-weighted phylogenetic diversity (PD_W), correlated against unweighted (NTI, upper panels) and abundance-weighted nearest taxon index (NTI_W, lower panels), from bacterial ASV data for all frequency levels and time points evaluated in this study (m = 184). Kendall correlation τ- and P-values adjusted at 5% FDR are indicated within the panels. Blue line represents locally estimated scatterplot smoothing regression (loess) with confidence interval in dark-grey shading. Note the inverted y-axis for both NTI and NTI_W, as values closer to zero indicate a higher relative contribution of stochastic assembly. Shaded in grey is the zone of significant stochastic phylogenetic dispersion, |NTI| < 2 and $|NTI_W| < 2$.

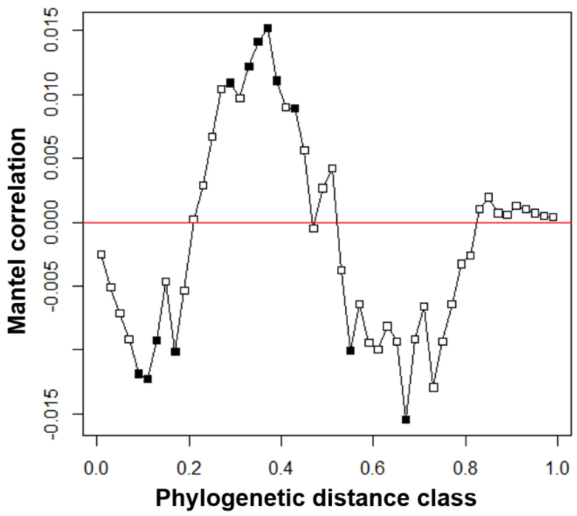

Supplementary Figure 5 - Phylogenetic Mantel correlogram evaluating phylogenetic signal in the sludge bioreactor communities sampled in this study. The plot relates between-ASV niche differences to between-ASV phylogenetic distances across a given phylogenetic distance. Significant correlations ($P_{adj} < 0.05$; closed symbols) indicate significant phylogenetic signal in ASV ecological niches within the associated phylogenetic distance class. The analysis shows a significant phylogenetic signal but mostly across relatively short phylogenetic distances.

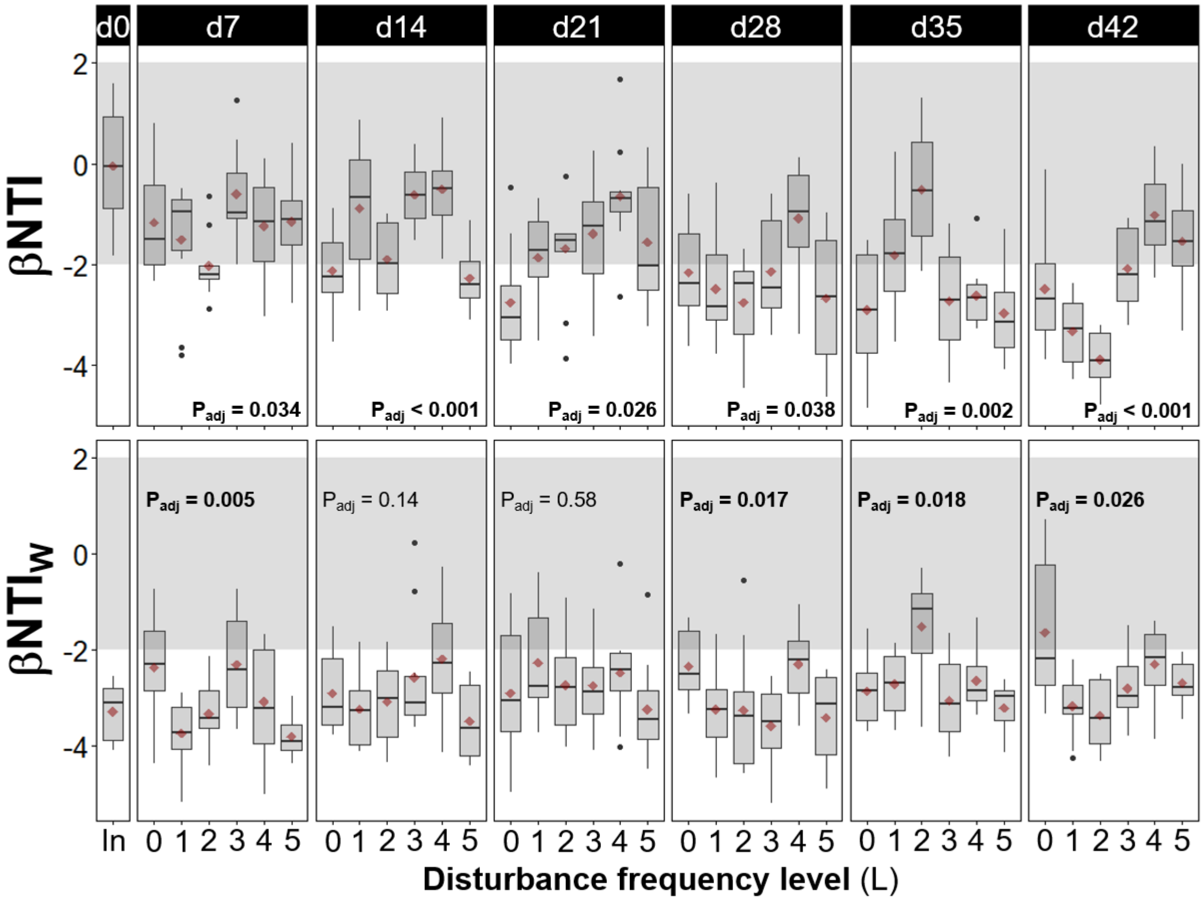

Supplementary Figure 6 – Temporal dynamics of community assembly via β-diversity null modelling of phylogenetic turnover across samples. Within-treatment pairwise values of the β-nearest taxon index, unweighted (βNTI, upper panels) and abundance-weighted (βNTI_W, lower panels), from bacterial ASV data for different frequencies of organic loading disturbance (n = 10). Disturbance frequency levels (L): 0 (undisturbed), 1-4 (intermediately disturbed), 5 (press disturbed). In: sludge inoculum (day 0, n = 6). Each panel represents a sampling day, red diamonds display mean values. Welch's ANOVA P-values adjusted at 5% FDR shown within panels. Shaded in grey is the zone where stochastic processes significantly dominate, |βNTI| < 2. βNTI values closer to zero indicate a higher relative contribution of stochastic assembly. The box bounds the interquartile range (IQR) divided by the median, and Tukey-style whiskers extend to a maximum of 1.5 times the IQR beyond the box.

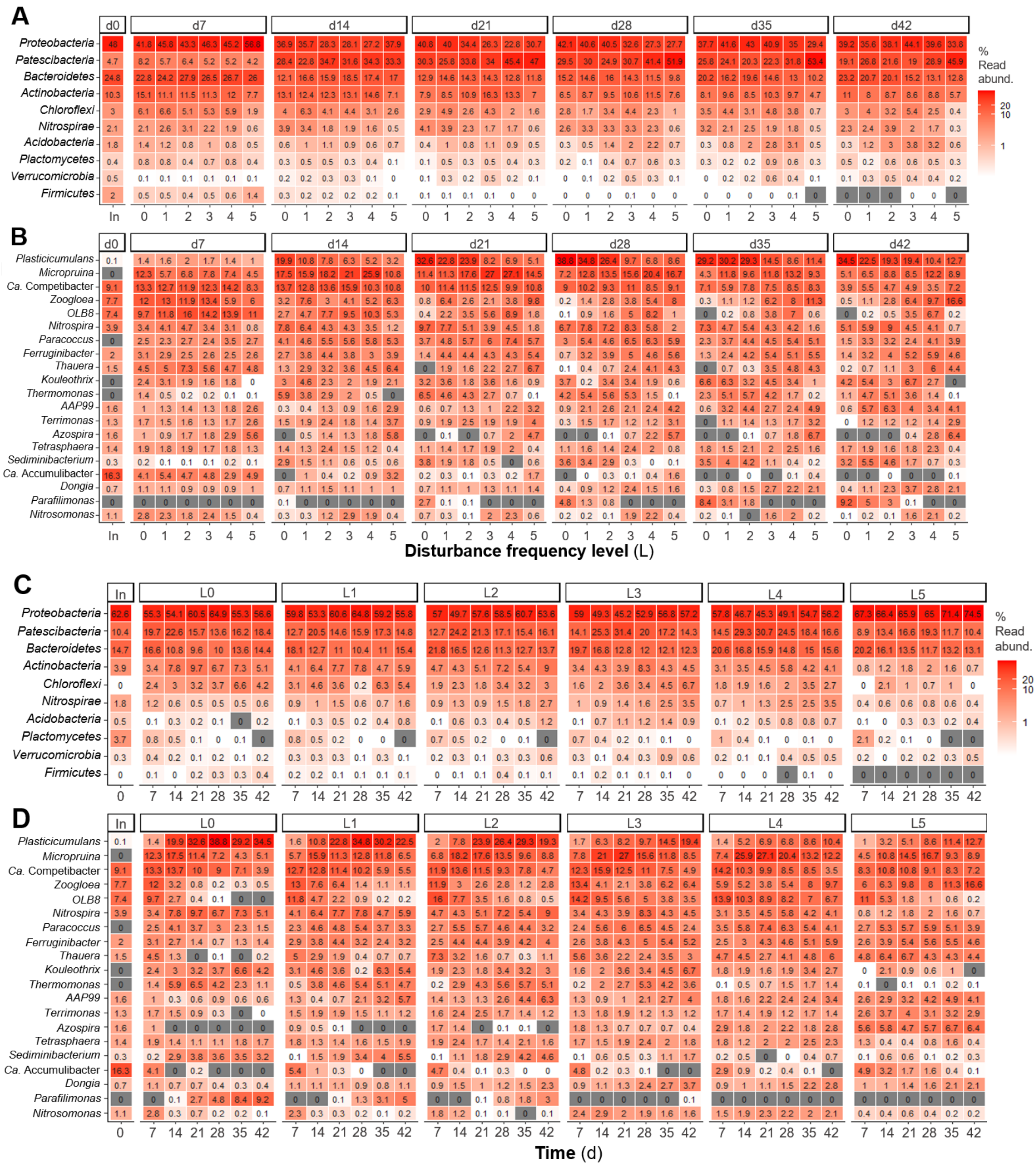

Supplementary Figure 7 – Community structure dynamics for bacterial (**A**, **C**) phyla and (**B**, **D**) genera, assessed through 16S rRNA gene metabarcoding. The 10 most abundant phyla and the 20 most abundant genera are shown (rows). Columns show the average percentage read abundance among reactors (n = 5) for (**A-B**) a given disturbance level across time, and for (**C-D**) a given day across disturbance levels. Disturbance frequency levels (L): 0 (undisturbed), 1-4 (intermediately disturbed), 5 (press disturbed). In: sludge inoculum (day 0, n = 4).

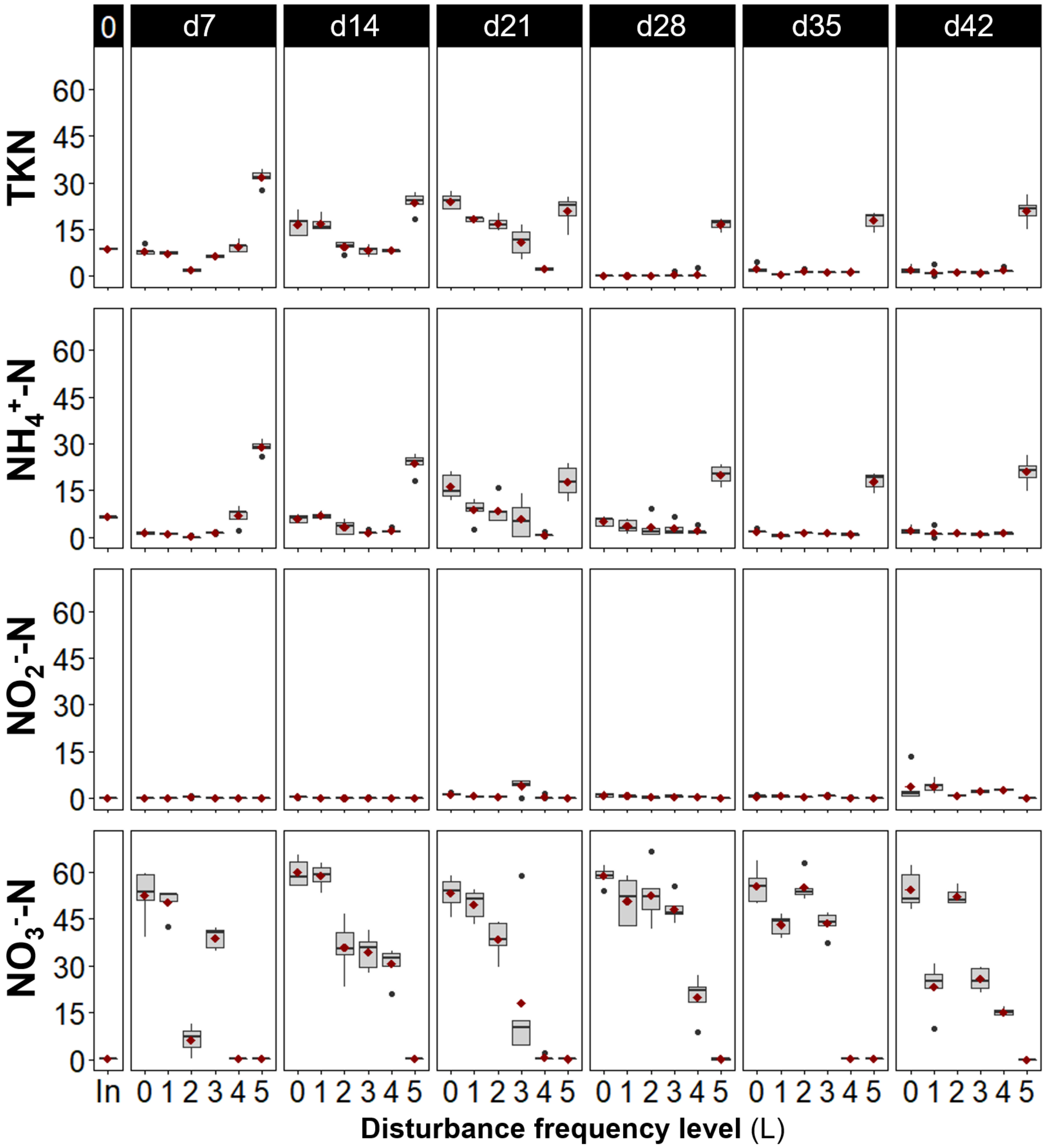

Supplementary Figure 8 – Effluent values of total Kjeldahl nitrogen (TKN), ammonia (NH_4^+-N), nitrite (NO_2^--N) and nitrate (NO_3^--N) as nitrogen for different frequencies of organic loading disturbance (n = 5). Disturbance frequency levels (L): 0 (undisturbed), 1-4 (intermediately disturbed), 5 (press disturbed). In: sludge inoculum (day 0, n = 4). Each panel represents a sampling day, and red diamonds display mean values. The box bounds the interquartile range (IQR) divided by the median, and Tukey-style whiskers extend to a maximum of 1.5 times the IQR beyond the box.

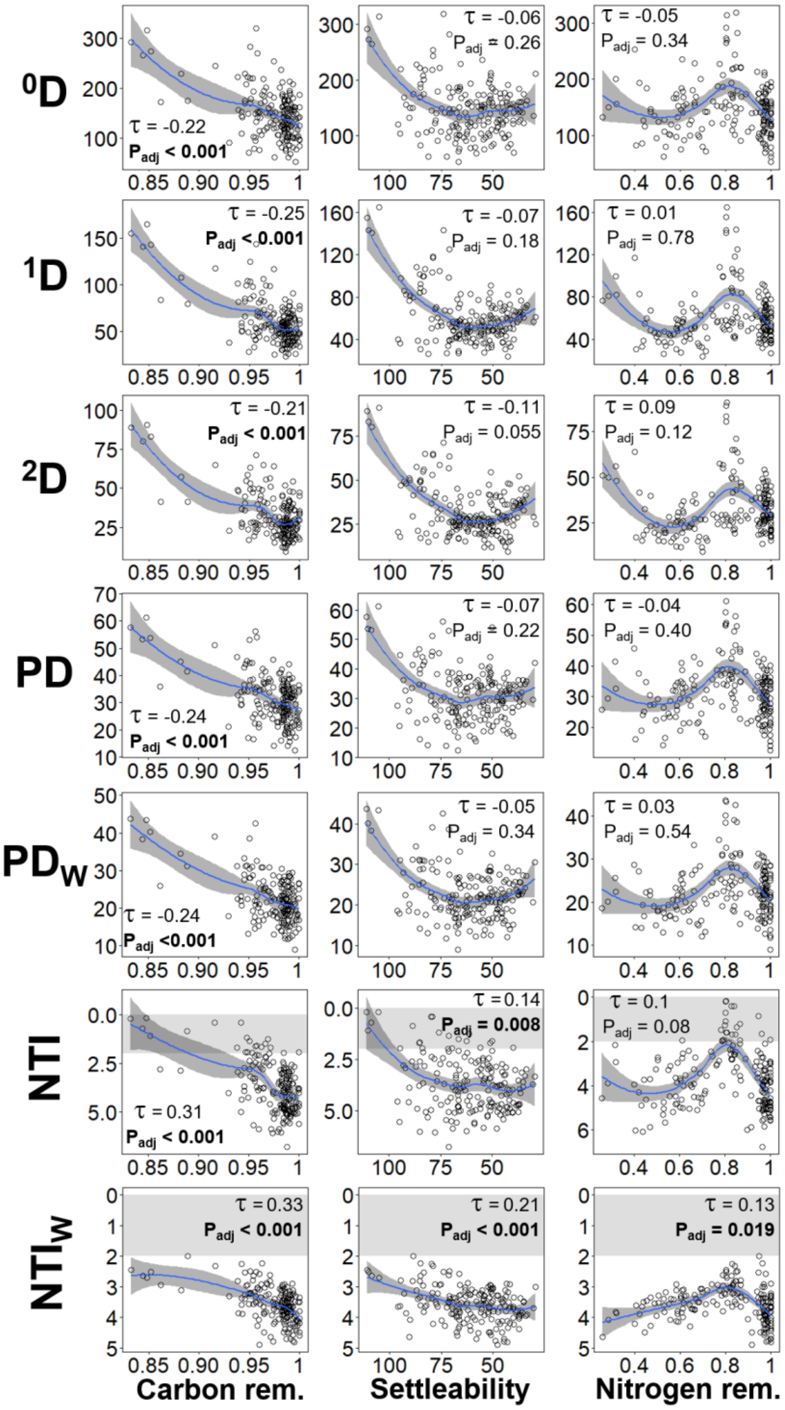

Supplementary Figure 9 – Community function assessed via influent chemical oxygen demand removal (carbon removal, left panels), sludge volume index (sludge settleability, middle panels), and influent total Kjeldahl nitrogen removal (nitrogen removal, right panels), correlated against richness (^{0}D), Hill α-diversity of 1st (^{1}D) and 2nd (^{2}D) order, unweighted (PD) and abundance-weighted phylogenetic diversity (PD_W), unweighted (NTI, upper panels) and abundance-weighted nearest taxon index (NTI_W, lower panels), from bacterial ASV data for all frequency levels and time points evaluated in this study (m = 184). Kendall correlation τ- and P-values adjusted at 5% FDR are indicated within the panels. Blue line represents locally estimated scatterplot smoothing regression (loess) with confidence interval in dark-grey shading. Shaded in grey is the zone of significant stochastic phylogenetic dispersion, $|NTI| < 2$ and $|NTI_W| < 2$. Note the inverted axis for sludge settleability, as it improves with decreasing SVI values, and for both NTI and NTI_W, since values closer to zero indicate a higher relative contribution of stochastic assembly.

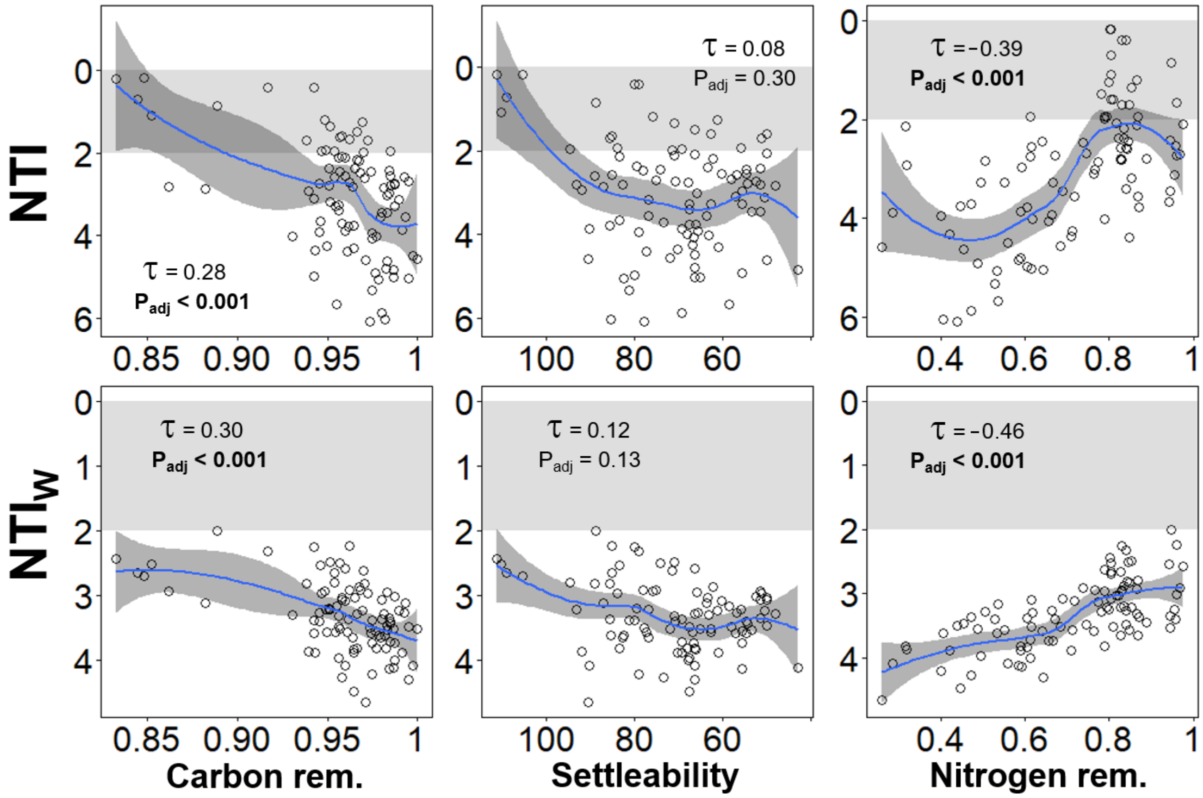

Supplementary Figure 10 – Community function assessed via influent chemical oxygen demand removal (carbon removal, left panels), sludge volume index (sludge settleability, middle panels), and influent total Kjeldahl nitrogen removal (nitrogen removal, right panels), correlated against unweighted (NTI, upper panels) and abundance-weighted nearest taxon index (NTI_W, lower panels), from bacterial ASV data at initial stages of succession (d0 to d21, m = 94). Kendall correlation τ- and P-values adjusted at 5% FDR are indicated within the panels. Blue line represents locally estimated scatterplot smoothing regression (loess) with confidence interval in dark-grey shading. Shaded in grey is the zone of significant stochastic phylogenetic dispersion, |NTI| < 2 and $|NTI_W| < 2$. Note the inverted axis for sludge settleability, as it improves with decreasing SVI values, and for both NTI and NTI_W, since values closer to zero indicate a higher relative contribution of stochastic assembly.

Level (Freq. of double org. loading)	Disturbance Frequency of organic loading shocks and sampling scheme [red X squares are High COD feeding days] [yellow squares are sampling days]																																											Pulses
0 (never)																																												0
1 (every 8 days)	x								x								x								x								x								x			6
2 (every 6 days)	x						x						x						x						x						x						x						x	8
3 (every 4 days)	x				x				x				x				x				x				x				x				x				x				x			11
4 (every 2 days)	x		x		x		x		x		x		x		x		x		x		x		x		x		x		x		x		x		x		x		x		x		x	22
5 (everyday)	x	x	x	x	x	x	x	x	x	x	x	x	x	x	x	x	x	x	x	x	x	x	x	x	x	x	x	x	x	x	x	x	x	x	x	x	x	x	x	x	x	x	x	43
Day	0	1	2	3	4	5	6	7	8	9	10	11	12	13	14	15	16	17	18	19	20	21	22	23	24	25	26	27	28	29	30	31	32	33	34	35	36	37	38	39	40	41	42	

Supplementary Figure 11 - Schematic representation of the experimental design and timeline, including disturbance frequency (left column) and incidence (right column). Red squares indicate double organic loading in the feed for that day. Level numbers ranged from 0 to 5 (0 for no disturbance, 1 to 5 for low to high disturbance frequency). Effluent and sludge sampling was done on the days marked in yellow.

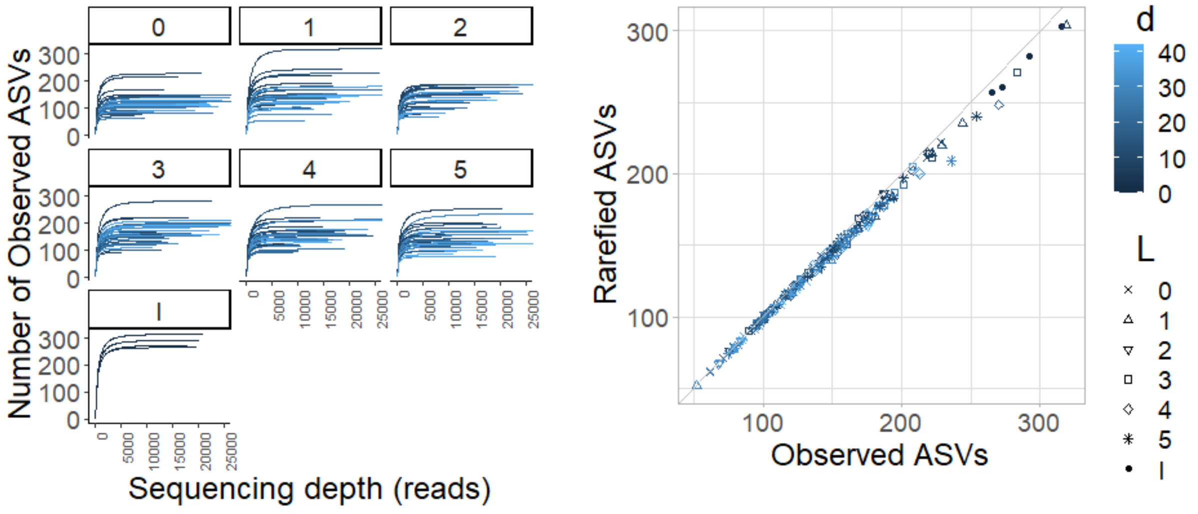

Supplementary Figure 12 – Rarefaction plots for 16S rRNA gene sequencing data. (A) Rarefaction curves for reactors within a given disturbance level across time. (B) Rarefied versus observed number of ASVs. Disturbance frequency levels (L, n = 5): 0 (undisturbed), 1-4 (intermediately disturbed), 5 (press disturbed). In: Sludge inoculum (day 0, n = 4).

Supplementary Table 1. Multivariate tests on bacterial community structure[Ψ] across disturbance frequency levels.

**Time** (d)	**No of levels***	***n*‡**	**df§ res**	**PERMANOVA†**		**PERMDISP†**	
				**F**	**P_{adj}¶**	**F**	**P_{adj}¶**
7	6	5	24	2.64	<0.001	1.44	0.50
14	6	5	24	5.19	<0.001	1.88	0.45
21	6	5	24	8.13	<0.001	2.66	0.13
28	6	5	24	9.75	<0.001	2.70	0.13
35	6	5	24	9.23	<0.001	0.67	0.92
42	6	5	24	11.9	<0.001	0.52	0.90

[Ψ]The Bray-Curtis dissimilarity metric was used on square-root transformed ASV relative abundance data.
* Factor levels (L): 0 (undisturbed), 1-4 (intermediately disturbed), 5 (press disturbed).
[†] Number of permutations used was 9,999
[‡] Number of replicates per level
[§] Degrees of freedom of the residual
[¶] P-values after correction for multiple comparisons at 5% FDR, via the Benjamini-Hochberg’s method.
