# Supplementary material for: Microbiome assembly predictably shapes diversity across a range of disturbance frequencies in experimental microcosms
Source: NPJ Biofilms Microbiomes. 2022 May 13;8:41. doi: 10.1038/s41522-022-00301-3 (PMC9106739; doi:10.1038/s41522-022-00301-3)
Supplement: Supplementary file 4 — NP Reporting Summary [file 41522_2022_301_MOESM4_ESM.pdf]

nature portfolio

Corresponding author(s): Stefan Wuertz

Last updated by author(s): Nov 15, 2021

Reporting Summary

Nature Portfolio wishes to improve the reproducibility of the work that we publish. This form provides structure for consistency and transparency in reporting. For further information on Nature Portfolio policies, see our Editorial Policies and the Editorial Policy Checklist.

Statistics

For all statistical analyses, confirm that the following items are present in the figure legend, table legend, main text, or Methods section.

n/a	Confirmed	
☐	☒	The exact sample size (*n*) for each experimental group/condition, given as a discrete number and unit of measurement
☐	☒	A statement on whether measurements were taken from distinct samples or whether the same sample was measured repeatedly
☐	☒	The statistical test(s) used AND whether they are one- or two-sided<br>*Only common tests should be described solely by name; describe more complex techniques in the Methods section.*
☐	☒	A description of all covariates tested
☐	☒	A description of any assumptions or corrections, such as tests of normality and adjustment for multiple comparisons
☐	☒	A full description of the statistical parameters including central tendency (e.g. means) or other basic estimates (e.g. regression coefficient) AND variation (e.g. standard deviation) or associated estimates of uncertainty (e.g. confidence intervals)
☐	☒	For null hypothesis testing, the test statistic (e.g. *F*, *t*, *r*) with confidence intervals, effect sizes, degrees of freedom and *P* value noted<br>*Give P values as exact values whenever suitable.*
☒	☐	For Bayesian analysis, information on the choice of priors and Markov chain Monte Carlo settings
☐	☒	For hierarchical and complex designs, identification of the appropriate level for tests and full reporting of outcomes
☐	☒	Estimates of effect sizes (e.g. Cohen's *d*, Pearson's *r*), indicating how they were calculated

Our web collection on statistics for biologists contains articles on many of the points above.

Software and code

Policy information about availability of computer code

Data collection	No software was used.
Data analysis	R-packages: dada2 (v.1.3.3), phyloseq (v.1.30.0), ggplot2 (v.3.3.2), ggdist (v.2.4.1), rstatix (v.0.6.0), ggpubr (v.0.4.0), ampvis2 (v.2.6.2), decipher (v.2.14.0), phangorn (v.2.5.5), metagMisc (v.0.0.4), picante (v.1.8.2), vegan (v.2.5.6). PRIMER (v.7) with PERMANOVA add-on. SILVA database (v.132).

For manuscripts utilizing custom algorithms or software that are central to the research but not yet described in published literature, software must be made available to editors and reviewers. We strongly encourage code deposition in a community repository (e.g. GitHub). See the Nature Portfolio guidelines for submitting code & software for further information.

Data

Policy information about availability of data

All manuscripts must include a data availability statement. This statement should provide the following information, where applicable:

- Accession codes, unique identifiers, or web links for publicly available datasets
- A description of any restrictions on data availability
- For clinical datasets or third party data, please ensure that the statement adheres to our policy

DNA sequencing data are available at NCBI BioProjects with accession number PRJNA723443. See supplementary information for details about the sludge inoculum collection, synthetic feed preparation, and additional figures of diversity and community assembly metrics, correlations, heat maps and data rarefaction. Diversity analyses on rarefied data and all other relevant data to reproduce the results of this study are available as supplementary files in the online version of this manuscript.

Field-specific reporting

Please select the one below that is the best fit for your research. If you are not sure, read the appropriate sections before making your selection.

☐ Life sciences ☐ Behavioural & social sciences ☒ Ecological, evolutionary & environmental sciences

For a reference copy of the document with all sections, see nature.com/documents/nr-reporting-summary-flat.pdf

Ecological, evolutionary & environmental sciences study design

All studies must disclose on these points even when the disclosure is negative.

Study description

The objective of this work was to test the central tenet of the ISH that intermediate disturbance frequencies promote stochastic assembly processes, resulting in increased alpha-diversity and variable beta-diversity. We employed 30 sequencing batch bioreactors at a microcosm scale (25-mL working volume), inoculated with activated sludge from a full-scale wastewater treatment plant in Singapore and operated for 42 days at 30°C in an incubator shaker. The daily complex synthetic feeding regime (adapted from Santillan et al. Ref. 37) included double organic loading at varying disturbance frequencies. Six levels of disturbance were set in quintuplicate independent reactors (n = 5), which received double organic loading either never (undisturbed), every eight, six, four, or two days (intermediately-disturbed), or every day (press-disturbed) (Fig. S11). Level numbers were assigned from 0 to 5 (0 for no disturbance, 1 to 5 for low to high disturbance frequency). Disturbance frequency was further calculated from the rate of high organic loading at each disturbance level resulting in values of 0, 1/8, 1/6, 1/4, 1/2, and 1. The number of double organic loading events at each disturbance frequency level (i.e., disturbance incidence) during the 42 days of the study were 0, 6, 8, 11, 22, and 42 (Fig. S11).

Research sample

The experiment was designed with six treatment levels of one factor with five replicates per treatment level (n = 5, total number of reactors was 6 x 5 = 30). The factor was double organic loading addition at different frequencies. Each of the 30 reactors was designed as a biological independent unit and all lab work practices were done to safeguard the independence of these reactors and avoid cross-contaminations.

Sampling strategy

Samples were taken from reactors for process performance parameters and microbial community analysis as described below in "Timing and spatial scale" section. The experiment was designed with six treatment levels of double organic loading addition frequencies with five replicate and independent reactors at each level so as to increase the power of the test.

Data collection

Data collection and recording was done by E. Santillan. The data collection procedure is thoroughly described in the Methods and Supplementary Materials and Methods sections. Parameters collected and the frequency at which they were collected is described in the next "Timing and spatial scale" section below.

Timing and spatial scale

Sludge samples of 2 mL (m = 184) were collected on the initial day of the study (four samples, taken at random from the inoculum mix) and weekly from each reactor afterwards (180 samples), for DNA extraction as previously described (Ref. 48). Ecosystem function, in the form of process performance parameters at the end of a cycle, was measured weekly in accordance with Standard Methods (Ref. 64) where appropriate, and targeted the removal of soluble COD and TKN from the mixed liquor after feeding. Sludge settling capacity was measured via the SVI (mL/g), considering 30 minutes of settling time. Concentrations in the mixed liquor of the bioreactors after feeding (i.e, beginning of a new cycle) were regularly 305.8 (±7.4) mg COD/L and 45.6 (±0.8) mg TKN/L, or 594.7 (±18.6) mg COD/L and 46.1 (±0.2) mg TKN/L when double organic loading occurred. A food-to-biomass ratio (F:M) control approach was used as previously described (Ref. 37), for which biomass was measured weekly as total suspended solids (TSS) after which sludge wastage was done to target a TSS of 1,500 mg/L. The latter resulted in average solids residence time (SRT) values of 30, 26, 23, 22, 19 and 15 days, for disturbance levels from 0 to 5, respectively. Note that these SRT values are well above the doubling times of relevant bacteria in activated sludge (Ref. 65). The duration of the experiment was set to 42 days due to clear signs of changes in ecosystem function across disturbance levels. Note that most relevant bacteria in activated sludge have generation times of less than 24 h. Hence, the 42-day length of this study represented around tens to hundredths of generations of many different taxa, allowing the detection of significant patterns in assembly and structure.

Data exclusions

Sequenced sample libraries were processed with the dada2 (v.1.3.3) R-package. Illumina adaptors and PCR primers were trimmed prior to quality filtering. Sequences were truncated after 280 and 255 nucleotides for forward and reverse reads, respectively. After truncation, reads with expected error rates higher than 3 and 5 for forward and reverse reads, respectively, were removed. After filtering, error rate learning, ASV inference and denoising, reads were merged with a minimum overlap of 20 bp. Chimeric sequences (0.17% on average) were identified and removed. For a total of 184 samples, an average of 18,086 reads were kept per sample after processing, representing 47% of the average forward input reads.

Reproducibility

The study was carried once using 30 independent 25-mL microcosm reactors which were subjected to six levels of disturbance with five replicates each, for 42 days. Replication was used as a measure of reproducibility in this study.

Randomization

Six levels of disturbance were set in five replicate independent reactors (n = 5, total number of reactors was 6 x 5 = 30), which received double organic loading every day (press-disturbed), every two, four, six, and eight days (intermediately-disturbed), and never (undisturbed). Tubes were numbered from 1 to 30 and the experimental units were randomly assigned among them. Level numbers were assigned from 0 to 5 (0 for no disturbance, 1 to 5 for low to high disturbance frequency) with different double organic loading addition frequencies employed.

Blinding

N/A

Did the study involve field work? ☐ Yes ☒ No

Reporting for specific materials, systems and methods

We require information from authors about some types of materials, experimental systems and methods used in many studies. Here, indicate whether each material, system or method listed is relevant to your study. If you are not sure if a list item applies to your research, read the appropriate section before selecting a response.

Materials & experimental systems

n/a	Involved in the study
☒	☐ Antibodies
☒	☐ Eukaryotic cell lines
☒	☐ Palaeontology and archaeology
☒	☐ Animals and other organisms
☒	☐ Human research participants
☒	☐ Clinical data
☒	☐ Dual use research of concern

Methods

n/a	Involved in the study
☒	☐ ChIP-seq
☒	☐ Flow cytometry
☒	☐ MRI-based neuroimaging
